# Detection of gene annotations and protein-protein interaction associated disorders through transitive relationships between integrated annotations

**DOI:** 10.1186/1471-2164-16-S6-S5

**Published:** 2015-06-01

**Authors:** Marco Masseroli, Arif Canakoglu, Massimiliano Quigliatti

**Affiliations:** 1 Dipartimento di Elettronica, Informazione e Bioingegneria, Politecnico di Milano, Piazza Leonardo da Vinci 32, 20133 Milano, Italy

## Abstract

**Background:**

Increasingly high amounts of heterogeneous and valuable controlled biomolecular annotations are available, but far from exhaustive and scattered in many databases. Several annotation integration and prediction approaches have been proposed, but these issues are still unsolved. We previously created a Genomic and Proteomic Knowledge Base (GPKB) that efficiently integrates many distributed biomolecular annotation and interaction data of several organisms, including 32,956,102 gene annotations, 273,522,470 protein annotations and 277,095 protein-protein interactions (PPIs).

**Results:**

By comprehensively leveraging transitive relationships defined by the numerous association data integrated in GPKB, we developed a software procedure that effectively detects and supplement consistent biomolecular annotations not present in the integrated sources. According to some defined logic rules, it does so only when the semantic type of data and of their relationships, as well as the cardinality of the relationships, allow identifying molecular biology compliant annotations. Thanks to controlled consistency and quality enforced on data integrated in GPKB, and to the procedures used to avoid error propagation during their automatic processing, we could reliably identify many annotations, which we integrated in GPKB. They comprise 3,144 gene to pathway and 21,942 gene to biological function annotations of many organisms, and 1,027 candidate associations between 317 genetic disorders and 782 human PPIs. Overall estimated recall and precision of our approach were 90.56 % and 96.61 %, respectively. Co-functional evaluation of genes with known function showed high functional similarity between genes with new detected and known annotation to the same pathway; considering also the new detected gene functional annotations enhanced such functional similarity, which resembled the one existing between genes known to be annotated to the same pathway. Strong evidence was also found in the literature for the candidate associations detected between *Cystic fibrosis *disorder and the PPIs between the *CFTR_HUMAN, DERL1_HUMAN, RNF5_HUMAN, AHSA1_HUMAN *and *GOPC_HUMAN *proteins, and between the *CHIP_HUMAN *and *HSP7C_HUMAN *proteins.

**Conclusions:**

Although identified gene annotations and PPI-genetic disorder candidate associations require biological validation, our approach intrinsically provides their *in silico *evidence based on available data. Public availability within the GPKB (http://www.bioinformatics.deib.polimi.it/GPKB/) of all identified and integrated annotations offers a valuable resource fostering new biomedical-molecular knowledge discoveries.

## Background

Continuous improvement of biotechnologies, progress of massive sequencing techniques and development of new technologies for high-throughput analysis and annotation of biomolecular sequences are generating a huge amount of biomolecular data and knowledge. Yet, the very valuable controlled biomolecular annotation data (i.e. the controlled descriptions of known characteristics of biomolecular entities, such as genes or proteins, through the association of the biomolecular entities with terms of a controlled vocabulary that describe such characteristics) are far from exhaustive.

To extract information and knowledge from available data, several approaches have been proposed. A literature-based knowledge discovery model has been first proposed by Swanson to identify implicit connections between terms that do not occur together in any scientific document [1]. Corpus-derived statistical models of semantic distance, such as Latent Semantic Analysis (LSA), have been evaluated as methods for the discovery of these implicit connections [2,3]. Other computational methods based on Singular Value Decomposition (SVD) of gene or protein annotation matrices have been developed to predict annotations [4,5]. Several approaches for link prediction in networks have been proposed [6]; mostly based on similarity algorithms and maximum likelihood or probabilistic models, they have been applied and evaluated mainly on social networks [7], but also in biology, particularly on protein-protein interaction data [8,9]. The use of decision trees and Bayesian networks for predicting annotations by learning patterns from available annotation profiles has been suggested as well [10]. Simpler yet effective logic rule techniques, such as the one based on transitive relationships [11], have been also proposed, in particular for their application to database relations [12]. Yet, huge efforts keep being performed to solve this issue and try to provide new biomolecular annotations reliably identified, which can complement the available ones and support uncovering new biomedical knowledge. Towards this aim, leveraging a high quality integration of available multiple heterogeneous, but consistent, information helps greatly.

Previously, we developed the Genomic and Proteomic Knowledge Base (GPKB) [13], an updated public, high-quality and consistent integration of reconciled heterogeneous and distributed annotation and interaction data; it can be profitably leveraged to help unveiling new biomedical knowledge by reliably identifying and supplementing missing annotations based on available ones. Here, we present and discuss our work aimed at 1) developing an efficient and automatic procedure to be routinely applied on new releases of the GPKB in order to detect consistent and trustworthy biomolecular annotations which are not present in the available data integrated, and 2) supplementing and providing them publicly, together with the available annotation and interaction data integrated in the GPKB, in support of biomedical knowledge discovery applications.

The data warehousing integration approach that we applied to build the GPKB allows performing thorough data quality and consistency checking [14], as well as reconciliation of unsynchronized data, in order to integrate only high quality consistent data [13]; both these aspects are paramount to subsequently use the integrated data for reliable comprehensive detection and supplement of missing biomolecular annotations. Furthermore, we drastically reduced warehousing maintenance overhead by using automatic procedures, which regularly update easily the data in the GPKB, and by adopting a novel, modular and multilevel feature-based global data schema [13]; besides easing data warehousing updates and extensions, it also ensures provenance tracking of all the integrated data, which is paramount for their proper subsequent processing and the interpretation of processing results.

Our developed annotation identification approach is inspired by the Swanson work [1], but founded on the transitive relationship logic rule [11]; in fact, it leverages the transitive relationships of heterogeneous extensive annotation data. Thus, it does not use a predictive model or provide predictions, but rather it detects and supplements annotations that should exist based on the available data. The applied concept is also close to the Linked Open Data approach of the Semantic Web [15], which has been recently used to link various sources of drug data in order to answer interesting scientific and business questions [16]. Yet, we enriched it with a set of novel rules that strengthen our approach and ensure its application only when the semantic type of the considered data and the semantic type and cardinality of their relationships allow identifying molecular biology compliant associations (see *Methods *section). This enhances the reliability of the detected annotations, which is further increased by the several procedures that we defined to avoid propagation, through automatic data processing, of errors existing in public biomolecular data, including in those that our method uses. The application of our approach to the high quality, consistent and reconciled data integrated in the GPKB allowed detecting and supplementing many missing new biomolecular annotations, "transferring" them from available ones. Validation of the transferred annotations showed their high reliability, which makes them suitable to be used for data-driven biomedical knowledge discoveries.

## Results

### Transitive relationship approach for biomolecular annotations

We implemented a general and customizable software framework to automatically detect missing biomolecular annotations and "transfer" them from available ones by transitive relationships based on available annotations, as defined in the *Methods *section and Additional file 1. It can be used with any biomolecular database that stores annotation data to perform the transitive relationship approach on large annotation data sets efficiently and effectively. Furthermore, it can automatically detect any meaningful semantic annotation, according to the defined set of novel rules illustrated in the *Transitive relationship approach and its defined rules *section of the *Methods *and to additional specific data attributes available; these last can be useful, for example, to maximize correctness and quality of the identified annotations, as discussed in the *Methods *in the *Control of error propagation during transitive relationship automatic approach *section.

**Figure 1:**
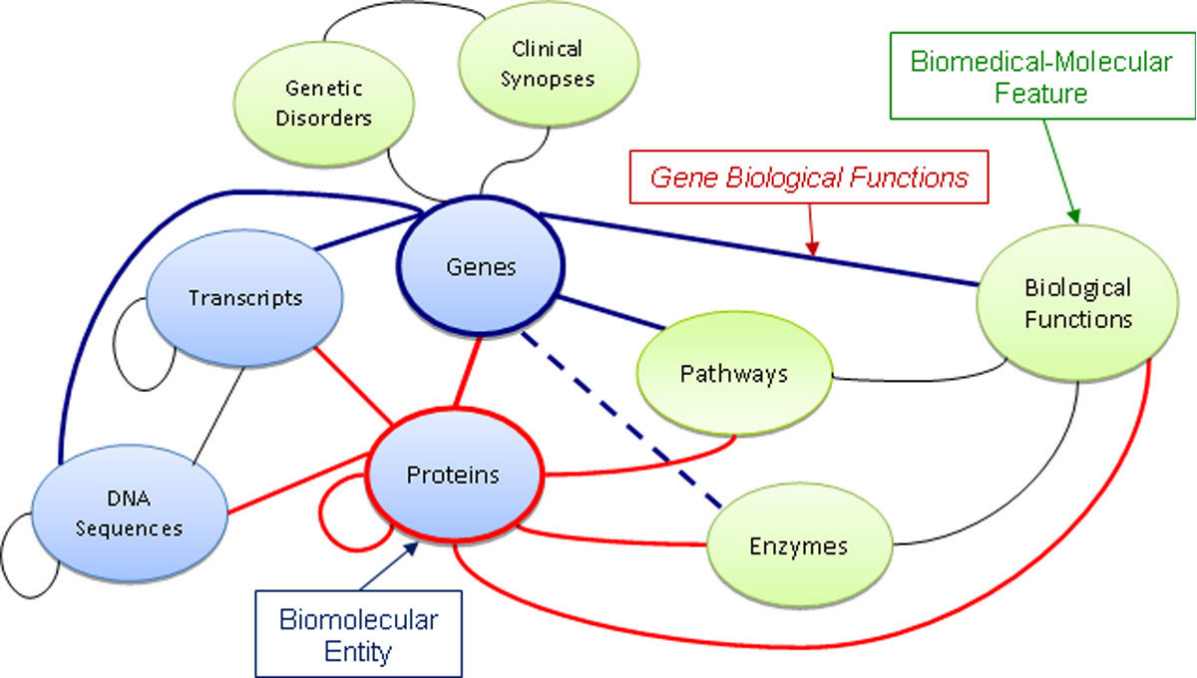
**GPKB feature network and protein annotation types considered for transitive relationship based transfer of annotations**. Solid line: types of available and transferred annotations; dotted line: types of only transferred annotations; bold red lines: types of available protein annotations considered for annotation transfer; bold blue lines: types of gene annotations transferred.

We focused mainly on transitive relationships with path of length two and used the developed software framework to detect and supplement missing new biomolecular annotations, according to the numerous gene and protein annotation and interaction data integrated in our GPKB (Table 1). Such data define a valuable network of many types of biomolecular entities, biomedical-molecular characteristics and their relationships. Figure 1 describes, at conceptual level, this network, which can be profitably leveraged by the transitive relationship method in order to discover and supplement missing annotations, transferring them from available ones. In Figure 1, each node of the network indicates a type of feature (i.e. biomolecular entity, or biomedical-molecular characteristic) whose data are in the GPKB; it represents a database table containing all instances of that feature (e.g. all genes, or all biological functions) in the GPKB. Similarly, each arc of the network indicates a relationship between the two connected features, defined by the annotation data in the GPKB; it represents a database feature association table that contains all the associations in the GPKB between the two connected features (e.g. all gene biological function annotations, or gene to protein associations), which can be of a single or multiple semantic types. (Notice that some of these semantic types define directed associations, while other express symmetric ones; thus, in Figure 1 each arc is shown undirected since it represents multiple associations of different semantic types.)

**Table 1:** Biomolecular entities, PPIs and annotations with biomedical-molecular characteristics integrated in the Genomic and Proteomic Knowledge Base.

	# of Items (*Homo sapiens*)	# of Organisms	Total Annotations (*Homo sapiens*)	Gene Annotations (*Homo sapiens*)	Protein Annotations (*Homo sapiens*)
**DNA Sequences**	563,760 (18,712)	12,904	-		
**Genes**	16,199,505 (47,487)	14,221	32,956,102 (348,662)		
**Transcripts**	8,065,827 (106,509)	406	-		
**Proteins**	56,990,212 (97,749)	477,175	273,522,470 (744,729)		
**PPIs**	277,095 (63,488)	1,073	-		-
**Enzymes**	5,403	7	220,964 (3,155)	-	220,964 (3,155)
**Biological Functions** **(Gene Ontology Terms)**	41,285	479,950	306,032,538 (1,012,297)	32,841,035 (304,911)	273,191,503 (707,386)
**Biochemical Pathways**	29,459	28	211,526 (64,395)	101,523 (30,207)	110,003 (34,188)
**Genetic Disorders**	7,853	1	13,430 (13,430)	13,430 (13,430)	-
**Clinical Synopses**	63	1	114 (114)	114 (114)	-

Depending on semantics of features and their associations, and on cardinality of associations, only some feature associations can be straightforwardly identified and transferred by transitive relationship based on available association data; we expressed these constrains in a set of novel rules described in the *Transitive relationship approach and its defined rules *section of the *Methods*. Here as follows, only as brief trivial explanatory example, we describe, in term of biological entities and annotations, which associations can be identified and transferred by transitive relationship and which not.

If a protein P_1 _(e.g. human *Breast cancer type 1 susceptibility protein *(*BRCA1_HUMAN*), or any of its isoforms) is ANNOTATED TO a biological function B_1 _(e.g. *Regulation of transcription from RNA polymerase III promoter*), then also the gene G_1 _(e.g. human *Breast cancer 1, early onset *(*BRCA1*)), which ENCODES the protein P_1 _and its isoforms, should be ANNOTATED TO the biological function B_1_. If such annotation of gene G_1 _to the biological function B_1 _is not available, but the annotations of P_1 _to B_1 _and G_1 _to P_1 _are available, then the annotation of G_1 _to B_1 _can be straightforwardly detected as missing and transferred by transitive relationship, with path of length two, based on available annotation data. Whereas, if both gene G_2 _and protein P_2 _are ANNOTATED TO the same biological function B_2_, it does not imply that gene G_2 _should ENCODE protein P_2_, given the possible multiple cardinality of annotation of unrelated genes and proteins to the same biological function. As well, if gene G_2 _ENCODES protein P_2 _and is ANNOTATED TO biological function B_2 _(as well as to many other biological functions), it does not straightforwardly imply that protein P_2 _should be ANNOTATED TO biological function B_2_; in fact, by alternative splicing, gene G_2 _could encode multiple proteins (besides P_2_) with different biological functions. We note that this last is a conservative rule for annotation transfer (in fact, e.g. in the UniProt database, usually annotations are assigned to the main protein entry, which includes all the protein isoforms, and it is rarely clear to which protein isoform is associated each annotation). We adopt this rule to take well into account the underlying molecular biology and avoid annotation automatic transfers that might generate false positive annotations, despite losing possible correct ones. In Figure 1, there are represented the types of gene annotations (bold blue arcs) that we detected as missing and transferred by transitive relationship based on the types of protein annotations available in the GPKB (bold red arcs). Although transitive relationships need directed links, Figure 1 does not show directed arcs since it represents not only the annotations used for the transitive relationship method, but the entire GPKB feature network previously mentioned, which includes all the annotations, of various semantic types, integrated in the GPKB.

**Figure 2:**
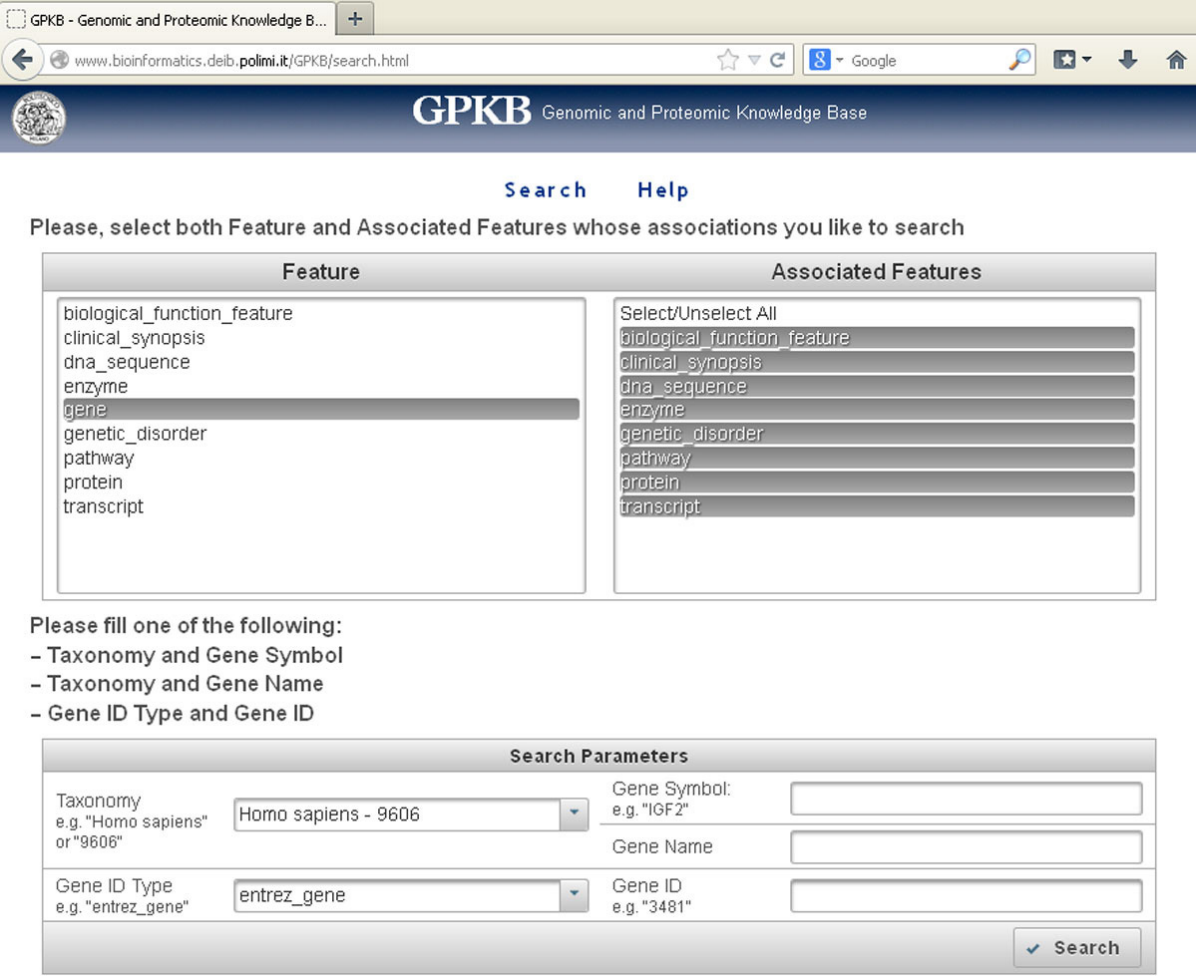
**GPKB Web interface: Search page**. Through an intuitive Web interface, the user can search and retrieve any of the annotations downloaded from multiple well known databases, or transferred by transitive relationship, which are integrated in the GPKB.

**Figure 3:**
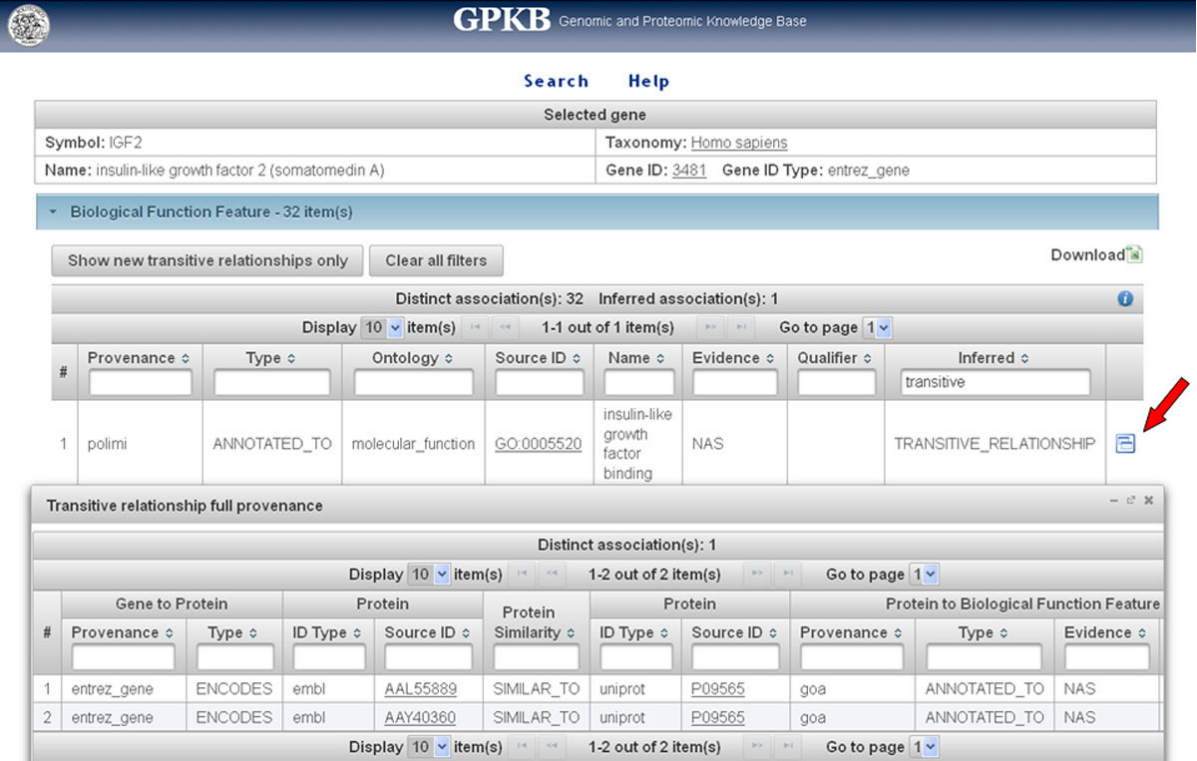
**GPKB Web interface: Search result page**. The transferred new annotation of the *Insulin-like growth factor 2 (somatomedin A) *(*IGF2*) human gene to the *Insulin-like growth factor binding *biological function is shown. (Notice the external links on all IDs, the "*Show new transitive relationships only*" button and the "*Download*" icon.) As all annotations in the GPKB that are transferred by the transitive relationship method, it is clearly marked with the value "*TRANSITIVE_RELATIONSHIP*" of its *Inferred *attribute. By clicking on the icon next to it (see the arrow), the "*Transitive relationship full provenance*" window pops up and shows all the available known association data on which the transitive relationship transfer was based. In the example shown, they are the *ENCODES *associations (provided by the Entrez Gene database) of the *IGF2 *human gene with the proteins with *AAL55889 *or *AAY40360 *EMBL ID, which are both associated by similarity with the protein with *P09565 *UniProt AC that (according to the GOA database) is annotated to the *Insulin-like growth factor binding *biological function with NAS (Non-traceable Author Statement) evidence.

Table 2 illustrates the quantity of annotations transferred by transitive relationship, as well as of the feature items and annotations available in the GPKB on which the transfer is based. All annotations transferred, which are not present in the data from the public databases integrated in the GPKB, have been stored in the GPKB; there, they are clearly identifiable as such based on the value (*TRANSITIVE_RELATIONSHIP*) of the *Inferred *attribute present in all GPKB annotation tables [13]. At http://www.bioinformatics.deib.polimi.it/GPKB/ they can be publicly searched, browsed and downloaded through the *GPKB *Web interface (Figure 2 and Figure 3). In particular, the gene annotations transferred (236,391 in total, 4,467 regarding *Homo sapiens*) were 20.68 % (2.72 % for *Homo sapiens*) of the same types of known gene annotations in the GPKB on which the annotation transfer was based (1,143,173 in total, 164,366 regarding *Homo sapiens*). As expected, fewer annotations are transferred in percentage for more studied organisms, such as *Homo sapiens*. Interestingly, the transferred gene annotations to pathways and biological functions were respectively 3.20 % and 2.10 % of the same type of annotations in the GPKB on which the annotation transfer was based. Different reasons may exist because such relevant gene annotations were not available in the important gene annotation data sources that we integrated in GPKB.

**Table 2:** Annotations transferred by transitive relationship and related feature items and annotations integrated in the GPKB on which the transfer is based.

# of Distinct Feature A Items Available (*Homo s*.)	# of Distinct Feature B Items Available (*Homo s*.)	# of Distinct Feature C Items Available (*Homo s*.)	# of Distinct Feature A / Feature B Annotations Available (*Homo s*.)	# of Distinct Feature B / Feature C Annotations Available (*Homo s*.)	# of Distinct Feature A / Feature C Annotations Available (*Homo s*.)	# of Distinct Feature A / Feature C Annotations Available Transferred (*Homo s*.)	% of Distinct Feature A / Feature C Annotations Available Transferred (*Homo s*.)
Genes: 14,848,524 (20,492)	Proteins: 11,736,361 (20,130)	Pathways: 513	12,031,396 (29,536)	104,416 (32,991)	98,316 (29,860)	3,144 (795)	3.20 % (2.66 %)
Genes: 14,848,524 (20,492)	Proteins: 11,736,361 (20,130)	Biological Functions: 41,285	12,031,396 (29,536)	704,382 (92,043)	1,044,857 (134,506)	21,942 (478)	2.10 % (0.35 %)
Genes: 14,848,524 (20,492)	Proteins: 11,736,361 (20,130)	Enzymes: 5,403	12,031,396 (29,536)	200,964 (3,155)	-	211,305 (3,194)	ALL
Genes: 14,848,524 (20,492)	Proteins: 11,736,361 (20,130)	Transcripts: 8,065,827 (106,509)	12,031,396 (29,536)	80,680 (31,463)	7,644,482 (80,964)	6,793 (1,262)	0.09 % (1.56 %)
Genes: 14,848,524 (20,492)	Proteins: 11,736,361 (20,130)	DNA Sequences: 563,760 (18,712)	12,031,396 (29,536)	163,396 (79,251)	16,107,408 (128,167)	7,690 (1,039)	0.05 % (0.81 %)
Proteins: 11,736,361 (20,130)	Genes: 14,848,524 (20,492)	Genetic Disorders: 7,853	12,031,396 (29,536)	12,013 (12,013)	-	15,344 (15,344)	ALL
PPIs 277,095 (63,488)	Genes: 14,848,524 (20,492)	Genetic Disorders: 7,853	50,863 (9,922)	12,013 (12,013)	-	1,027 (1,027)	ALL

Percentages of annotations transferred are with respect to annotations of the same type available in the GPKB; ALL: only annotations transferred, no annotations available in the GPKB.

Several (19.31 %) of the transferred gene to pathway annotations involve Reactome pathways, although such transferred annotations concern only human genes (since only for *Homo sapiens *Reactome provides protein annotations not computationally inferred). Despite Reactome provides pathway annotations for both proteins and genes, inconsistencies and not complete correspondences exist between such gene and protein annotations, which our approach detected and (partially) completed; we reported them to the Reactome curators, who ensured to fix them in the next release of their data. Also 2,537 (80.69 %) gene to KEGG pathway annotations were transferred, but for 12 organisms; most of these annotations, as well as of the transferred gene to biological function annotations, regards less studied organisms. They do not just fill gaps between databases, but represent new discovered gene annotations, which are transferred thanks to the protein similarity data integrated in the GPKB [13] and their use by the transitive relationship method (see an example in Figure 3 and its description in the *Example of relevant annotation transferred by transitive relationship *subsection below).

By leveraging gene associated disorder data from the OMIM database, we also identified possible candidate protein annotations to human genetic disorders (Table 2). To our knowledge, these annotations are not available in public databases. Furthermore, by taking advantage also of protein-protein interaction (PPI) data integrated in the GPKB from the IntAct database, we identified interacting proteins possibly associated with the same genetic disorder; in so doing, we detected 1,027 potential candidate associations of 317 genetic disorders with 782 human PPIs. All these are to be intended as proteins and PPIs candidate associated with genetic disorders, which are suggested for further association studies.

### Evaluation of the transitive relationship approach for biomolecular annotations

Differently from other proposed methods [2-10], which are based on predictive models and provide probabilistic predictions, the simple, yet effective, transitive relationship approach is based on logic rules [11]. Thus, it does not provide predictions; rather it gives discrete answers (positive/negative) in detecting and transferring those biomolecular annotations that should exist based on the available data. Classical model validation methods (e.g. k-fold cross-validation or Receiving Operator Characteristic (ROC) curves) are suitable to validate probabilistic but not discrete results [17], which are represented by a single point in the ROC space. Accuracy of the discrete results provided by the transitive relationship approach only depends on completeness and correctness of available data on which the approach is applied. For this reason we applied it on the numerous high quality reconciled data integrated in the GPKB, which can ensure better detection and supplement of missing biomolecular annotations.

To evaluate the transitive relationship approach, we estimated its *recall *(i.e. true positive rate, or sensitivity) and *precision *(i.e. true negative rate, or positive predicted value); we did so by comparing the gene annotations in the GPKB with the gene annotations that the approach identifies that should exist and can transfer based only on the protein annotation and protein encoding gene data available in the GPKB. (Notice that the gene annotations in the GPKB are not considered in such transitive relationship based annotation identification; they are only used for comparison with the identification results.) Overall, we obtained a recall of 90.56 % (99.09 %, 48.65 %, 99.03 % and 99.97 % recall for the gene to pathway, gene to biological function, gene to transcript and gene to DNA sequence annotations, respectively). The missed identification of some available gene annotations was mainly due to no availability of the corresponding protein annotations, or of data about the genes encoding the annotated proteins. Lower recall for biological function annotations was mainly due to the numerous of these annotations that are available as computationally derived only, both for genes and proteins; thus, they are available but our method does not considered them for annotation transfer to avoid possible automatic error propagation (see *Methods *section).

As estimate of method precision, overall we found that 96.61 % of the gene annotations that the transitive relationship method identified were already available in the GPKB (99.46 % gene to pathway, 75.70 % gene to biological function, 99.24 % gene to transcript and 99.66 % gene to DNA sequence annotations, respectively). Yet, as the available annotations are incomplete by definition, in particular for the many less studied organisms considered, these good figures can only represent a possible approximation of the method precision and not its correct estimate.

### Assessment of transferred biomolecular annotations

As discussed in the previous section, classical validation methods cannot be used to assess the transitive relationship method results. Thus, to better evaluate their correctness, we performed an overall co-functional evaluation of all genes involved in the transferred and known gene pathway annotations, as well as in known and transferred biological functions. In addition, we performed a supervised *in silico *validation and biological interpretation of some annotations transferred in some selected biological examples. We performed the latter one by consulting the literature and several well-known databases and by taking advantage of the evidence, based on the available data, that our implemented approach provides; in fact, it keeps track and offers full characterization and provenance of all features and their associations involved in each of the new annotations transferred (e.g. see the "*Transitive relationship full provenance*" window in Figure 3).

**Figure 4:**
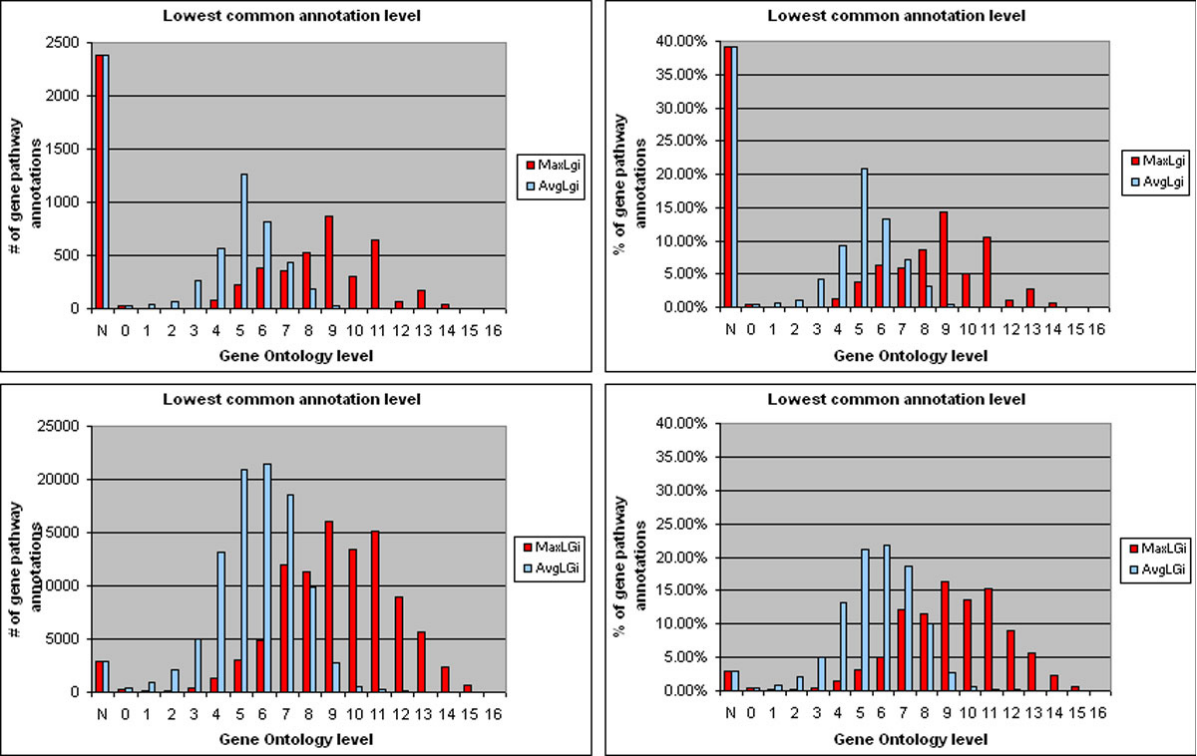
**Co-functional evaluation of genes with pathway annotation transferred by transitive relationship and with known GO annotation**. Upper histograms, MaxLg_i _and AvgLg_i_: maximum and average of the levels in the Gene Ontology (GO) hierarchy of the lowest common known GO functional annotations shared between each gene with transferred annotation to a pathway and the genes known to be involved in that pathway; lower histograms, MaxLG_j _and AvgLG_j_: as MaxLg_i _and AvgLg_i _respectively, but between each gene known to be involved in a pathway with transferred gene annotation and all the other genes known to be involved in that pathway; GO level 0 pertains to ontology root shared annotation, higher GO levels pertain to more specific shared GO annotations; level category N represents gene pathway annotations (transferred or known) whose gene does not have any GO annotation.

***Co-functional assessment of genes with transferred and known pathway and biological function annotations***. By performing the co-functional evaluation of these genes as described in the *Methods *section, we obtained the results illustrated in Figure 4. The simple idea behind this global co-functional evaluation is that a pathway annotation is correctly transferred to a gene if the gene has biological function (i.e. Gene Ontology - GO) annotations similar to the GO annotations of the genes already known to be annotated to the same pathway. The transferred pathway annotations of genes with known GO functional annotations resulted to be 60.85 % of all gene pathway annotations transferred. About 90.86 % of them regards genes whose most specific (lowest common) GO annotation shared with the genes known to be involved to the same pathway has maximum level (MaxLg_i_), in the GO hierarchy, higher than level 5, i.e. it is quite or very specific (upper histograms in Figure 4). Also on average the shared lowest common GO functional annotations are rather specific; in 89.22 % of the gene pathway annotations transferred their average GO level (AvgLg_i_) is higher than level 3.

**Figure 5:**
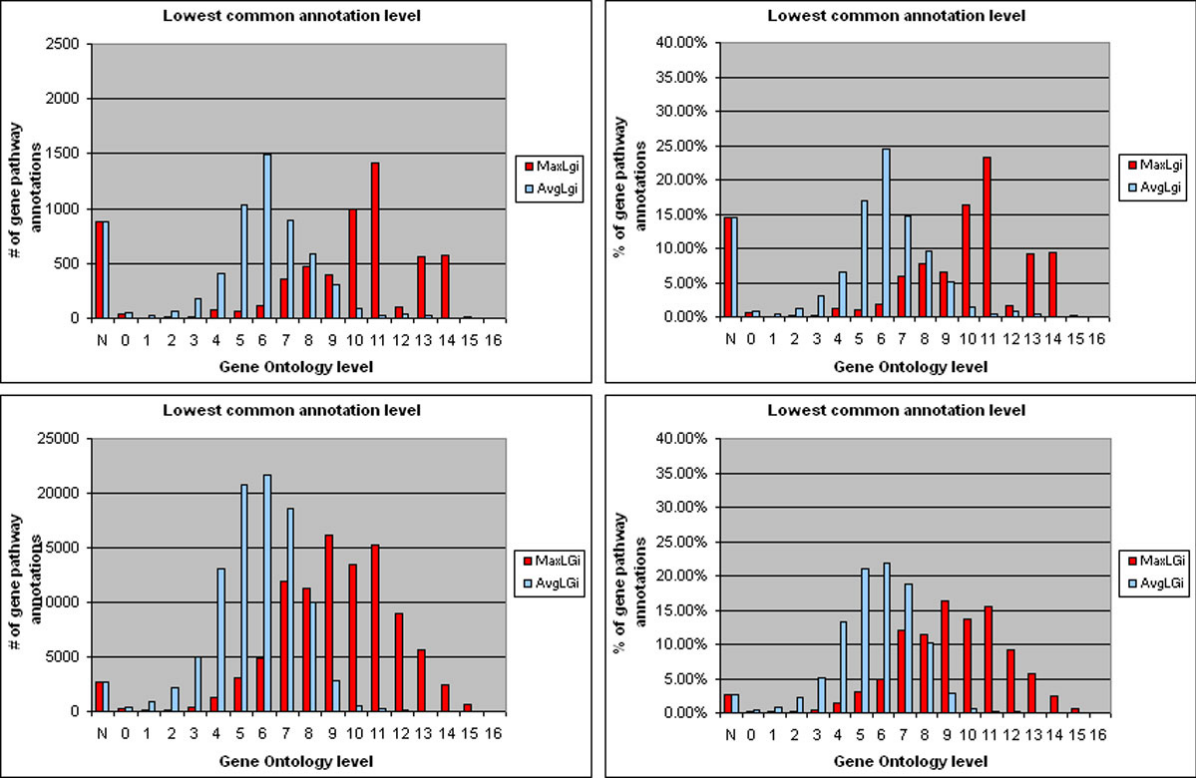
**Co-functional evaluation of genes with pathway annotation transferred by transitive relationship and with known or transferred GO annotation**. Same as Figure 4, but obtained by considering also the GO functional annotations transferred to the genes by transitive relationship, instead of only the gene known GO functional annotations. In so doing, more than half of the genes without known functional annotation results having specific transferred GO functional annotation(s), i.e. with high GO level.

Most important, these percentages are very similar to the equivalent ones obtained for the known pathway annotations of genes known to share GO functional annotations with other genes known to be involved in the same pathway. For 94.31 % of them, MaxLG_j _is higher than GO level 5, while for 91.31 % of them AvgLG_j _is higher than GO level 3 (lower histograms in Figure 4). Although these known gene pathway annotations, which regard genes with known GO annotations, are a higher percentage, out of all known pathway annotations available, with respect to the percentage of transferred gene pathway annotations (97.02 % vs. 60.85 %), this is expected. To a certain extent, pathway and GO functional annotations are related; thus, it is expected that genes with known pathway annotations have more known GO annotations. By considering not only the known GO annotations but also the ones that we transferred by transitive relationship, the percentage of pathway annotations transferred to genes with GO annotations raises from 60.85 % to 85.57 %. Furthermore, the distributions of MaxLg_i _and AvgLg_i _enhance, with increased values for high GO levels (96.17 % vs. 90.86 % of genes with MaxLg_i _higher than GO level 5 and 93.61 % vs. 89.22 % of genes with AvgLg_i _higher than GO level 3), while the distributions of MaxLG_j _and AvgLG_j _values remain similar (Figure 5). Conversely, for the known pathway annotations of genes known to share GO functional annotations with other genes known to be involved in the same pathway, all these values practically do not change by considering also the GO annotations that we transferred by transitive relationship.

These results clearly show that, in the great majority of the pathway annotations transferred to genes, the annotated gene has function very similar to that of at least one of the genes known to be involved in that same pathway, and also similar on average to the functions of all those genes. Most of all, such considerations can be equally applied to the genes already known to be annotated to the same pathway. Furthermore, a relevant part of the residual gene pathway annotations transferred which seam without functional evidence are due to the incompleteness of the available gene functional annotations. In fact, by considering also the new gene functional annotations transferred through the transitive relationship method, this residual percentage of transferred gene pathway annotations lowers to more than half (14.43 %), which is closer to the one of the known gene pathway annotations available (2.98 %). This also shows the relevance and reliability of the transferred gene to biological function annotations.

**Figure 6:**
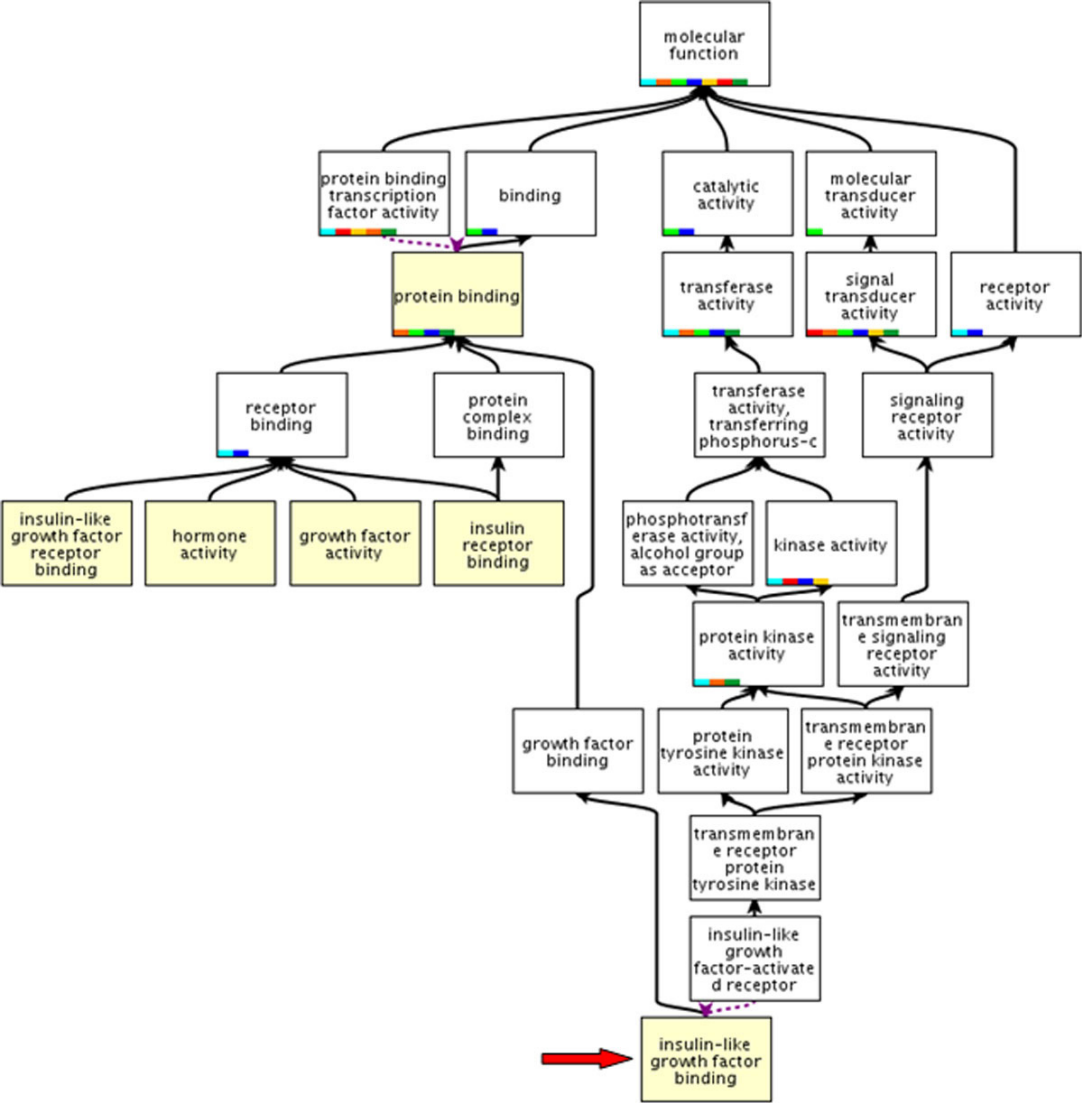
**Some Gene Ontology annotations available for the *IGF2 *human gene and its new detected one**. Yellow upper boxes represent five of the most specific Gene Ontology molecular function annotations available for the *Insulin-like growth factor 2 (somatomedin A) *(*IGF2*) human gene; the arrow indicates the new detected annotation.

***Example of relevant annotation transferred by transitive relationship***. As an example of the ability of our, although simple, transitive relationship based method to discover non-trivial gene annotations, we report the detection of the new annotation of the *Insulin-like growth factor 2 (somatomedin A) *(*IGF2*) human gene to the GO *Insulin-like growth factor binding *molecular function. It is transferred from the same annotation (with "Non-traceable Author Statement (NAS)" evidence) of the Swiss-Prot reviewed human *Putative insulin-like growth factor 2-associated protein *(*IG2R_HUMAN*). In major databases this protein is not defined as encoded by the *IGF2 *human gene, which results encoding the *Insulin-like growth factor II *(*IGF2_HUMAN*) protein and its isoforms. Such isoforms do not include the *IG2R_HUMAN *protein, although since 1988 a paper describes it as one of the alternative splicing forms encoded by the *IGF2 *human gene [18]. Correctness of the new annotation is also supported by, and in agreement with, other GO annotations already available for the *IGF2 *human gene (Figure 6). In particular, it is not contradictory with the *insulin like growth factor receptor binding *annotation known for the *IGF2 *human gene; in fact, the *IGF2 *human gene encodes both the two alternative splicing forms above mentioned [18], which have different binding affinity within the IGF signaling regulatory system. The detection of this non-trivial gene annotation (i.e. not directly coming from the annotations of a protein explicitly known as encoded by the gene) leverages the power not only of the transitive relationship method, but also of the protein similarity data integrated in the GPKB [13]. In fact, these data include the association of the *IG2R_HUMAN *protein (P09565 UniProt AC) with the AAL55889 and AAY40360 EMBL/GenBank protein IDs, which are also associated with the *IGF2 *human gene; thus, they transitively associate the *IG2R_HUMAN *protein with the *IGF2 *human gene as another of its encoded isoforms (Figure 3). The subsequent application of the transitive relationship method to such unveiled ENCODES relationships and the ANNOTATED TO relationships between the *IG2R_HUMAN *protein and its GO annotations allows identifying and transfer the *Insulin-like growth factor binding *as a new GO annotation for the *IGF2 *human gene (Figure 3).

**Figure 7:**
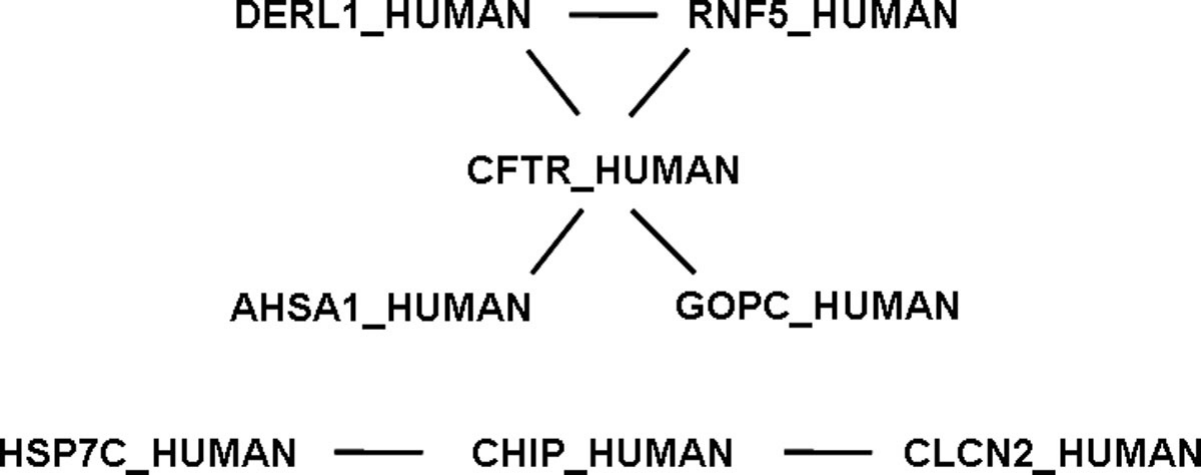
**The seven pairwise interactions, between eight human proteins, that have been detected as candidate associated with the *Cystic fibrosis *genetic disorder**.

***Assessment of PPI-genetic disorder candidate associations***. Unfortunately, it is not possible performing a global evaluation also for the identified candidate associations between PPIs and genetic disorders; in fact, no information to globally compare them with is available, although a few papers exist about the involvement of disrupted PPIs in the onset and development of some genetic disorders (e.g. [19]). Thus, our final validation could regard only some of the 1,027 potential associations detected between 782 human PPIs and 317 major genetic disorders (Additional file 2). Among these latter ones, we focused on the long studied *Cystic fibrosis*, one of the common inherited diseases in humans; we found strong evidence in the literature that supports six out of the seven identified candidate associations of *Cystic fibrosis *with the PPIs illustrated in Figure 7. In fact, those PPIs include the interactions of the *CFTR_HUMAN *protein with the four *DERL1_HUMAN, RNF5_HUMAN, AHSA1_HUMAN *and *GOPC_HUMAN *proteins, as well as the interactions of the *CHIP_HUMAN *protein with the *HSP7C_HUMAN *and *CLCN2_HUMAN *proteins. Mutations of the encoding genes of all these proteins, in particular of the *Cystic fibrosis transmembrane conductance regulator *(*CFTR*) human gene, as well as of many other genes (50 in total), are individually known to be directly involved in different grades and manifestations of *Cystic fibrosis*, which arises from misfolding and premature degradation of mutated *CFTR *forms. In addition, Younger et al. [20] discovered an endoplasmic reticulum membrane-associated ubiquitin ligase complex that cooperates with the cytosolic *HSP7C */ *CHIP *E3 complex and contains interacting protein products of the *DERL1 *and *RNF5 *genes, which cooperate to triage variants of the *CFTR *protein in order to monitor their folding status and promote proteasomal degradation. In 2006, Wang and colleagues [21] observed that the down-regulation of human *Hsp90 cochaperone AHA1 *(*AHSA1*) rescues misfolding of *CFTR *protein in *Cystic fibrosis*. Lately, they characterized the molecular and structural basis of the mechanisms responsible for such regulation [22], thus providing a potential key to understanding the role of *Hsp90 *in folding of *CFTR *and progression of *Cystic fibrosis *disease. More recently, Pelaseyed and Hansson [23] elucidated the modulated down-expression of *CFTR *through over-expression of *GOPC*, which directs *CFTR *for degradation. All these works support and provide evidence for six of the candidate associations identified between *Cystic fibrosis *and the six PPIs mentioned. We could not find clear supporting evidence only for the identified candidate association of *Cystic fibrosis *with the PPI between the *CHIP_HUMAN *and *CLCN2_HUMAN *proteins, although also the latter protein is known to be associated with *Cystic fibrosis*, since it is over-expressed in epithelia affected by *Cystic fibrosis *[24].

All the identified candidate associations could suggest that some types of the *Cystic fibrosis *multi-variant disorder may be associated with defects in the interactions between these proteins. In the review [19], Zanzoni et al. previously reported that gene mutations may alter the interaction properties of the encoded proteins, disrupting the interaction interface and leading to loss of function and disorders. They also suggested that PPIs could represent a class of targetable entities for novel therapeutic strategies. Possibly, in *Cystic fibrosis *different mutations could alter the functional interaction of the *CFTR_HUMAN *protein with the *DERL1_HUMAN, RNF5_HUMAN, AHSA1_HUMAN *and *GOPC_HUMAN *proteins, or between the *CHIP_HUMAN *and *HSP7C_HUMAN *or *CLCN2_HUMAN *proteins. If this would be experimentally confirmed, such finding could also suggest, as a possible disease treatment strategy, the engineering of a synthetic protein interacting, e.g., with the mutated *CFTR_HUMAN *protein and similar in function to the *DERL1_HUMAN, RNF5_HUMAN, AHSA1_HUMAN *or *GOPC_HUMAN *protein, whose interaction with the mutated *CFTR_HUMAN *protein results altered. At the time of writing, to our knowledge, such associations of *Cystic fibrosis *with the mentioned PPIs were not reported in any public database or explicitly in the literature; making them available in the GPKB represents an important advance.

## Discussion

Obtained results clearly show that the simple, yet effective, transitive relationship technique [11] can be originally applied to discover gene annotations from data structured in a large biomedical-molecular database, such as the GPKB. Our implementation, optimized for its off-line use, allows obtaining results in reasonable time, also when it is applied on huge amounts of genomic and proteomic data. Furthermore, when it is required, it can be further accelerated by using it in a distributed and parallel way on partitioned data. We are not aware of any large biomolecular database that currently implements such transitive relationship procedure, which produces results of great benefit to the community, as we showed.

The major issue in the use of the transitive relationship approach is in the possible propagation of errors, i.e. the generation of wrong results due to incorrectness of the considered data or of their semantic types. Being very conscious of this aspect, we implemented numerous mechanisms to avoid wrong results; during both GPKB construction and transitive relationship processing, these mechanisms exclude data items that must not be taken into account, or of low quality. In particular, we leveraged our previous efforts in the identification of errors and inconsistencies in public databases [14] by taking maximum care in avoiding error propagation through erroneous transitive relationships due to asynchronisms, inconsistencies, or errors in the considered data [25-28]. Whenever possible, we identified inconsistencies, reconciled asynchronisms and excluded erroneous data from the transitive relationship approach, as described in the *Methods *section. Furthermore, we adopted several precautions to ensure biological significance of results. Firstly, we considered only transitive relationships with path of length two, avoiding full transitive closure. Secondly, we focused only on biomedical-molecular relationships whose semantic type and cardinality allow straightforward transitive relationships, as formalized in a set of novel rules that we defined in the *Methods *section. The only exception was the identification of PPIs candidate associated with genetic disorders, which is based on the available association data between genetic disorders and genes encoding the interacting proteins. In this case, only potential candidate associations can be detected, since for alternative splicing a gene can encode multiple proteins with different characteristics. Nonetheless, given the high relevance of such candidate associations, which presently to our knowledge are not available in any public database, we decided to detect them anyway, although knowing that the results could include errors and that there is a long way from associating interacting proteins to a single disease and the disruption of their interaction to this disease. However, we could find strong supporting evidence in the literature for the few detected candidate associations that we validated, i.e. the ones detected between *Cystic fibrosis *and human PPIs.

Validation of results obtained through the implemented method demonstrated that the transitive relationship approach, applied on the numerous and heterogeneous annotation data integrated in the GPKB, can clearly detect and supplement missing gene annotations which are already implicitly known as annotations of the gene encoded proteins; in so doing, not only it fills gaps and fixes inconsistencies among different data sources, but also it makes explicit relevant information useful for computational analyses. Furthermore and much more significantly, it can also reliably identify and supplement substantial novel gene annotations, as shown in a non-trivial example in the *Results *section; this is possible mainly thanks to the data quality checking and integration procedures performed during the GPKB construction and the historical and similarity data integrated in the GPKB [13].

Despite the GPKB integrates all data publicly available on the FTP sites of the reference Entrez Gene and GOA databases, as well as of some relevant gene pathway databases (i.e. BioCyc, KEGG and Reactome), by using the transitive relationship approach we could detect and transfer numerous gene pathway and biological function (Gene Ontology) annotations not included in these databases. Co-functional evaluation of genes with transferred and known annotation to the same pathway, demonstrated overall reliability of the transferred gene pathway, as well as biological function, annotations.

All obtained results demonstrate the usefulness of our approach in reliably identifying new annotations, as well as in complementing the ones provided by individual databases. This is particularly relevant for the gene annotations to characteristics of their encoded proteins, which are still limitedly provided by on-line available databases. Indeed, gene annotations are very important, in particular for the interpretation of routinely performed transcriptomic analyses; the great majority of the many tools available for functional enrichment analysis of gene expression results directly relies on the available gene annotations [29]. Improvement of such gene annotations in quantity, coverage and quality is paramount to obtain better gene functional analysis results. Our approach and its application to any new release of the GPKB provide a relevant contribution towards this aim.

Public availability in the GPKB of all new annotations detected complements our work in the construction of the GPKB as an updated integrated collection of relevant biomedical-molecular data sparsely available. It makes the GPKB an even more valuable data source for integrated bio-searches on several types of annotation data, aimed at answering complex biomedical questions that can lead to biomedical knowledge discovery, as we showed in [30]. Furthermore, the provenance tracking implemented in GPKB allows users to exactly know the origin of each data integrated in the GPKB, as well as of all the annotations detect by transitive relationship and the data used for their detection. This enables each user to independently assess quality and confidence in the data, as well as to select and use only those data that he/she considers more reliable.

In the next scheduled release of the GPKB, besides the detected gene annotations and PPI-genetic disorder potential candidate associations, we plan to include also transcript annotations identified based on available annotations of the proteins that the transcripts encode. Subsequently, we intend to enrich the GPKB also with DNA sequence annotations detected on the basis of available annotations of genes and proteins encoded by the DNA sequences. Presently, both DNA sequence and transcript annotations are not directly available in any public database, forcing the researchers interested on them to tedious and error prone conversions from available gene and protein annotations. Their public availability would ease and improve biomedical interpretation of different types of high-throughput biomolecular experimental results, including those that are recently obtaining with DNA-seq and RNA-seq next generation sequencing techniques.

## Conclusions

Biomolecular annotations can be efficiently and effectively detected *in silico *by leveraging integrated data from multiple databases through a transitive relationship based approach. The detected annotations require biological validation; yet, this approach intrinsically provides their *in silico *evidence based on the available data. Evaluation of obtained results demonstrated that this approach can correctly detect with good precision not only annotations that are already present in some databases on which the transitive relationship approach is not based, but also new valuable annotations not yet included in any database. In the former case, the same annotations available in some database validate the detected ones; in the latter case, we evaluated some of the new identified annotations and found relevant scientific papers that support them. Their public availability can improve bioinformatics analyses that are carried out by using available biomedical-molecular annotations. Their storage in the GPKB, together with the available annotations there integrated, allows leveraging the GPKB to perform integrated bio-searches, which may foster data-driven discoveries that can help unveiling new biomedical-molecular knowledge.

## Methods

### Available biomolecular annotations considered

We applied and tested our developed transitive relationship based approach on the very numerous, heterogeneous, high-quality and reconciled annotation and interaction data integrated in the GPKB (Table 1); they regard biomolecular entities (i.e. DNA sequences, genes, transcripts and proteins) and their associations with many different biomedical-molecular characteristics, e.g. biological functions (i.e. GO biological processes, molecular functions and cellular components), biochemical pathways, genetic disorders and clinical synopses. At the time of writing, the GPKB integrated all these data, as well as molecular interaction data, downloaded the last time on September 1^st ^2014 from several well known databases, carefully selected according to their renowned relevance and reliability; they included Entrez Gene, UniProt, IntAct, Expasy Enzyme, GO, GOA, BioCyc, KEGG, Reactome and OMIM (whenever possible we integrate in GPKB data retrieved from their original provider, since in this case they are supposed to be the most reliable data available). The great amount of high-quality heterogeneous biomolecular association data integrated in the GPKB makes it a unique valuable resource where performing comprehensive evaluations on all the integrated data that it contains. Thus, we used the GPKB as database where to apply and test the implemented transitive relationship method to detect missing biomedical-molecular annotations.

### Transitive relationship approach and its defined rules

Depending on its semantic type, the relationship between single items (i.e. with cardinality 1 to 1) can own or not the transitive property. This property states that, if for all items A, B and C an item A is related to an item B (A → B) and the item B is related to an item C (B → C), then by transitive relationship also the item A is related to the item C (A → C) [11]. Yet, when multiple items are related to one another, even if the relationship semantic type holds the transitive property, it does or does not provide meaningful results by transitive relationship depending on the cardinality (1 to n, or n to n) of the relationship. In particular, if an item B is related to multiple items (A_i (i: 1-n) _and C_j (j: 1-m)_), then it does not straightforwardly mean that all such items are related to each other (i.e. all A_i _are related to all C_j_, as well as all A_i _are related to each other and all C_j _are also related to each other). Some items A_i _could be related to some, or even all, items C_j _and *vice versa*, but a global relationship (which would have n to n cardinality) between all items A_i (i: 1-n) _and C_j (j: 1-m) _can not be derived. However, even if an item B is related to multiple items C_j (j: 1-m)_, if the item B is related to a single item A, then it directly and meaningfully implies that such item A is related to all items C_j (j: 1-m) _(A → C_j (j: 1-m)_), i.e. with a relationship with cardinality 1 to n.

We applied the above considerations to the relationships described by existing biomolecular annotation data in order to detect missing annotations, by transitive relationship based on the available annotations, and transfer them from existing annotations. First, we classified the semantic types of these relationships and their cardinality, according to the semantic type of the related items and their underlying molecular biology (taking into account that in the annotation data what are related are the IDs of the related items). Thus, for example, the cardinality of the relationship between DNA sequence and protein is always 1:1, or 1:n if alternative splicing occurs. In fact, paralog DNA sequences have different IDs as well as their encoded paralog proteins. Then, depending on such semantic types and cardinalities, we defined the possible semantic types of the biologically meaningful biomolecular annotations that can be transferred by transitive relationship. The items involved in biomolecular annotations can be biomolecular entities (i.e. DNA sequences, genes, transcripts and proteins), or biomedical-molecular characteristics (e.g. biological functions, biochemical pathways, genetic disorders, etc.). Such item semantic types are always clearly defined in available annotation data; furthermore, their correctness for the data integrated in the GPKB is carefully and thoroughly controlled by the GPKB data quality and consistency checking procedures used. The semantic types of the relationships between such items, described by available biomolecular annotations, can be summarized as follows. The semantic type of a relationship between two biomolecular entities can be more generic (i.e. RELATED_TO), or more specific (i.e. ENCODES, INTERACT_WITH). The relationship between two biomedical-molecular characteristics is usually generic (i.e. ASSOCIATED_WITH), as well as the semantic type between a biomolecular entity and a biomedical-molecular characteristic (i.e. ANNOTATED_TO), unless in the latter case the biomedical-molecular characteristic represents a molecule directly interacting with the biomolecular entity; in this case, the semantic type of the relationship is INTERACT_WITH. Consequently, the semantic type of a relationship identified by transitive relationship over existing relationships of such semantic types can be always generically defined as ANNOTATED_TO, when the identified relationship is between a biomolecular entity and a biomedical-molecular characteristic. Similarly, when it is between two biomolecular entities, it can be a generic RELATED_TO, or a more specific ENCODES (when all involved relationships are of semantic type ENCODES), or INTERACT_WITH (when it regards biomolecular entities that encode interacting biomolecular entities, e.g. genes encoding interacting proteins). All such relationships usually have 1 to 1, or 1 to n cardinality; thus they can be straightforwardly and meaningfully transferred by transitive relationship. This would not be the case for relationships between biomedical-molecular characteristics, which would generally have n to n cardinality; thus, we did not transfer them by transitive relationship. Also for the transferred relationships, their cardinality is clearly defined by the related semantic types and the underlying biology (taking into account that what are related are the IDs of the biomolecular entities and biomedical-molecular characteristics). Thus, based on the rules defined above, we could reliably transfer protein annotations to the genes that encode the annotated proteins; furthermore, but only as possible candidate annotations suggested for further study, we could transfer genetic disorder annotations of genes to the gene encoded proteins and to the interactions of proteins (PPIs) encoded by genes annotated to the same genetic disorder.

### Control of error propagation during transitive relationship automatic approach

Since errors and inconsistencies exist in public biomolecular database data [25,26], automatic processing of these data can increase and propagate such errors and affect the correct identification of new annotations [27,28]. To avoid it, we implemented several control procedures devoted to ensure high reliability of identified annotations. First, we focused the transitive relationship approach only on those annotations with transitive semantic relationships and suitable cardinality (1 to 1, or 1 to n), as illustrated and discussed above in the *Transitive relationship approach and its defined rules *section. Second, we applied our approach only on quality checked and reconciled data, as the ones integrated in the GPKB [14]. Third, we avoided considering not current data (i.e. marked as obsolete by the data source that provides them), or transferring annotations that would be inconsistent with any of the available data attributes. For example, we did not transfer any biological functional annotation to genes classified as pseudogenes (i.e. non-functional genomic DNA sequences); we did so to avoid transferring annotations that could be incorrect, although knowing to miss some correct ones. In fact, we verified (data not shown) that in the public databases in some cases the pseudogene classification is not correct, or is assigned to genes that have both protein coding and pseudogene alleles, i.e. which are polymorphic genes. Examples of such genes are the olfactory receptor family members (e.g. *OR10J4, OR1P1, OR2J1, OR4E1, OR4K3, OR4Q2 *and *OR51J1*) whose allele biological variation can partially explain the different olfactory capabilities among subjects. Finally, from the transitive relationship approach we also excluded annotations derived only from previous automatic inferences, e.g. GO annotations of proteins provided by GOA, or pathway annotation of proteins from Reactome, with only evidence code "*Inferred from Electronic Annotations*" (*IEA*). A recent paper [31] shows that GO computationally inferred annotations of proteins have reached a quality that might be comparable to that of the GO curated annotations which are not based on experimental evidence; yet, we prefer not taking into account GO annotations with only IEA evidence, since they are usually considered less reliable than the other GO annotations (also the EMBL-EBI QuickGO tool provides separate evaluations of co-occurring GO terms based on non-IEA annotations only, e.g. http://www.ebi.ac.uk/QuickGO/GTerm?id=GO:0070531#term=stats). Accordingly, we also avoided to recursively consider, in subsequent transitive relationships, annotations derived in previous transitive relationship steps (e.g. a new annotation A → C that was transferred on the basis of a transitive relationship A → B → C is not considered in a transitive relationship A → C → D). Furthermore, to avoid redundancies, in the case of ontological annotations, we also checked if an annotation, which would be detected as missing by transitive relationship, is between a biomolecular entity and an ontology term that in the ontology structure is ancestor of a term already annotated to that biomolecular entity. In this case, we avoid detecting such annotation as missing by transitive relationship. In fact, for the ontological annotation inheritance property (which is also known as "true path rule" for the Gene Ontology annotations), such annotation is implicitly included in the annotations already available for that biomolecular entity.

### Co-functional assessment of genes with transferred and known pathway and biological function annotations

Reliability of the gene pathway annotations transferred through the transitive relationship approach was evaluated as follows. First, we extracted all the lowest common ancestors (LCAs) [32] between each of the known GO functional annotations of each gene g_i _with a transferred annotation to a pathway P and each of the known GO functional annotations of each gene G_j _known to be involved in P. We considered the level in the GO hierarchy of each of these LCAs (taking the higher level when a LCA has multiple GO levels) and calculated the maximum level (MaxLg_i_-G_j_) of the LCAs of each g_i_-G_j _gene pair. Next, for each gene g_i_, we computed the maximum (MaxLg_i_) and average (AvgLg_i_) of these MaxLg_i_-G_j _levels. MaxLg_i _and AvgLg_i _provide evidence of the specificity of the most specific functional feature shared between a gene with a transferred annotation to a pathway and at least one (MaxLg_i_), or all on average (AvgLg_i_), of the genes known to be involved in that pathway. The higher MaxLg_i _(and to a certain extent AvgLg_i_) is, the more evidence exists that supports the reliability of transferring the annotation to that pathway to gene g_i_. Furthermore, we compared such evidence with the equivalent one available for the genes known to be involved in a pathway. Towards this goal, we repeated the same described evaluation for each gene G_j_, by extracting the LCAs between each of its known GO annotations and each of the known GO annotations of each of the other genes G_j _known to be involved in the same pathway P. Then, for each gene G_j_, we likewise computed the maximum (MaxLG_j_) and average (AvgLG_j_) of the levels in the GO hierarchy of the lowest of these LCAs for each gene pair. Finally, we compared the distributions of quantity and percentage of pathway annotations transferred to genes g_i _(and of known pathway annotations of genes G_j_) over all MaxLg_i _(MaxLG_j_) and AvgLg_i _(AvgLG_j_) levels, respectively.

## List of abbreviations used

AHSA1: AHA1, activator of heat shock 90kDa (Hsp90) ATPase homolog 1 gene

BRCA1: Breast cancer 1, early onset gene

CFTR: Cystic fibrosis transmembrane conductance regulator gene

GO: Gene Ontology

GPKB: Genomic and Proteomic Knowledge Base

IEA: Inferred from Electronic Annotations

IG2R_HUMAN: Human putative insulin-like growth factor 2-associated protein

IGF2: Insulin-like growth factor 2 (somatomedin A) gene

IGF2_HUMAN: Human insulin-like growth factor II protein

IPI: Inferred from Physical Interaction

LCA: Lowest Common Ancestor

LSA: Latent Semantic Analysis

NAS: Non-traceable Author Statement

PPI: Protein-Protein Interaction

ROC: Receiving Operator Characteristic

SQL: Structured Query Language

SVD: Singular Value Decomposition

## Competing interests

The authors declare that they have no competing interests.

## Authors' contributions

MM conceived the project, was responsible for its supervision and coordination, was involved in the design and validation of the approach, and wrote this manuscript.

AC contributed to develop and test the approach; implemented, optimized and applied it to the data in the GPKB, obtaining and validating the here illustrated results, and contributed to write this manuscript.

MQ contributed to develop and test the approach, validated some of the obtained results, and contributed to write this manuscript.

## Supplementary Material

Additional file 1 **Implementation of the transitive relationship approach and benchmarking of its alternative SQL strategies**. Click here for file

Additional file 2 **Genetic disorders with potential associations with PPIs detected by transitive relationship**. Click here for file

## Declarations

This research work is part of, and has been partially supported by, the "Data-Driven Genomic Computing (GenData 2020)" PRIN project (2013-2015), funded by the Italian Ministry of the University and Research (MIUR), which also funded the publication of this article.

This article has been published as part of *BMC Genomics *Volume 16 Supplement 6, 2015: Proceedings of the Italian Society of Bioinformatics (BITS): Annual Meeting 2014: Genomics. The full contents of the supplement are available online at http://www.biomedcentral.com/bmcgenomics/supplements/16/S6.
