# Supplementary material for: Detection of gene annotations and protein-protein interaction associated disorders through transitive relationships between integrated annotations
Source: BMC Genomics. 2015 Jun 1;16(Suppl 6):S5. doi: 10.1186/1471-2164-16-S6-S5 (PMC4460591; doi:10.1186/1471-2164-16-S6-S5)
Supplement: Additional file 1 — Implementation of the transitive relationship approach and benchmarking of its alternative SQL strategies. [file 1471-2164-16-S6-S5-S1.pdf]

Additional file 1: Implementation of the transitive relationship approach and benchmarking of its alternative SQL strategies

Implementation of the transitive relationship approach

In order to automatically detect missing biomolecular annotations and "transfer" them from available ones by transitive relationship based on the available annotations, we implemented a general and customizable software framework using Java programming language and standard Structured Query Language (SQL); we did so since most of the available biomolecular annotations is stored in relational databases. Usually, a generic biomolecular database holds multiple instances of biomolecular entities and biomedical-molecular characteristics, and multiple types of their mutual relationships (i.e. biomolecular annotations). The latter ones are generally stored in appropriate feature association tables, which can be used to identify biomolecular annotations by transitive relationship [A1]. By using some paradigmatic feature association database tables with a few example data (including only the fundamental attributes with feature identifiers and with no association semantic types), Figure A1 shows the conceptual steps of the transitive relationship method when two feature associations are considered; the cases with more than three features involved and with specific association semantic types derive straightforwardly. The goal is discovering all possible distinct pairs of feature X_i and feature X_j identifiers, which are not already included in the database table featureX_i2featureX_j, by applying the transitive relationship approach on the data in all pairs of featureX_i2featureX_k and featureX_k2featureX_j tables available (for all i, j and k). The method proceeds in two

steps. Firstly, two related association data tables (e.g. featureA2featureB and featureB2featureC) are connected by INNER JOIN on their common attribute (featureB_id); this generates all possible triplets of identifiers of the three features in the two related feature association tables considered (featureA_id, featureB_id, featureC_id). Secondly, the association data table related to both association tables initially considered (featureA2featureC) is taken into account; if it is available, all triplets initially obtained that include the pairs of identifiers already contained in this last table ($featureA\_id_i$, $featureC\_id_j$) are removed from the firstly obtained data. The remaining triplets contain all the new associations discovered between the features in the last considered association table (i.e. between feature A and feature C). Although it would not be required, we decided to adopt the triplet data structure, which includes the identifier of the joining feature (featureB_id), in order to keep track of the origin of the identified associations. As well, we also store the types of the involved transitive relationships. All these data completely define the provenance of the detected annotations. Through them, as well as the complete provenance information of each of the data integrated in the GPKB from multiple sources, every user can assess from where and how each new identified annotation has been found, and thus which is its quality and confidence (Figure 3).

The first step of the transitive relationship method can be straightforwardly implemented in a relational database by using the following SQL statement, which stores the obtained results in the *ResTab* table:

CREATE TABLE ResTab AS SELECT … FROM featureA2featureB AS FAB

INNER JOIN featureB2featureC AS FBC USING (featureB_oid)

Conversely, the second step of the method can be implemented in SQL using three different strategies: I) by avoiding to generate all possible triplets of identifiers of the

three features in the two related feature association tables considered, and directly extracting only the identifier triplets that do not include pairs of feature identifiers already contained in the third association table considered; II) by deleting from the triplets of feature identifiers firstly generated the ones that include pairs of feature identifiers already contained in the third association table considered; III) by extracting only the identifier triplets of the features in the two association tables firstly joined that do not match with any entry in the third association table considered, when this last is connected by *LEFT JOIN* on the two attributes in common with the two association tables initially joined together. Thus, the transitive relationship method between two features can be globally implemented in SQL in the following three different ways, each one having different performances in terms of memory usage and running time:

Strategy I):

CREATE TABLE ResTab AS SELECT … FROM featureA2featureB AS FAB

INNER JOIN featureB2featureC AS FBC USING (featureB_oid)

WHERE (FAB.featureA_oid, FBC.featureC_oid) NOT IN

(SELECT featureA_oid, featureC_oid FROM featureA2featureC AS FAC);

Strategy II):

CREATE TABLE ResTab AS SELECT … FROM featureA2featureB AS FAB

INNER JOIN featureB2featureC AS FBC USING (featureB_oid);

DELETE FROM ResTab WHERE (featureA_oid, featureC_oid) IN

(SELECT featureA_oid, featureC_oid FROM featureA2featureC AS FAC);

Strategy III):

CREATE TABLE ResTab AS SELECT … FROM featureA2featureB AS FAB

INNER JOIN featureB2featureC AS FBC USING (featureB_oid)

LEFT JOIN featureA2featureC AS FAC USING (featureA_oid, featureC_oid)

WHERE FAC.featureA_oid IS NULL;

It is worth clarifying that for simplicity the above SQL statements of the three strategies do not include any additional constraint. All required constrains can be easily included in the SQL WHERE statement, e.g. to select relationships of specific type (as specified in the "*Transitive relationship approach and its defined rules*" section of the *Methods*), or to improve the quality of the identified relationships (as discussed in the "*Control of error propagation during transitive relationship automatic approach*" section of the *Methods*).

Since transitive relationships of biomolecular annotations can involve very numerous data, we benchmarked these three different strategies in order to implement the best performing one. It should be noticed that our aim was not to solve the efficient implementation of the full recursive transitive closure for all possible multiple feature combinations. Yet, it was to implement an efficient automatic transitive relationship based identification – also between multiple features – of only new biomolecular annotations that are molecular biology compliant and of best quality, based on the available annotations and our defined rules (see "*Transitive relationship approach and its defined rules*" section of the *Methods*). Although specific implementations of the transitive relationship approach can be done by using stored procedures, which are Data Base Management System (DBMS) specific, we did not consider this option since we aimed at an efficient standard SQL implementation usable in any relational DBMS. Thanks to its generality and customizability, our implementation of the transitive relationship approach can be used with any database that stores association data.

Benchmarking of alternative SQL strategies for the transitive relationship approach

In order to test and compare the performance of each of the three possible SQL strategies that can be used to implement the transitive relationship approach, we considered three generic features (feature A, feature B and feature C) and some random, but realistic, data describing their direct associations (i.e. between feature A and feature B, feature B and feature C, and feature A and feature C). Then, we tested the detection of new associations between each pair of such features by transitive relationship based on the considered association data. We performed all tests using a relational PostgreSQL 8.4 DBMS and using association data tables with the following characteristics:

- featureA2featureB table: 46,556,200 rows and 8 attributes
- featureB2featureC table: 6,000 rows and 7 attributes
- featureA2featureC table: 6,465 rows and 6 attributes

The schema of each association data table used was as follows: *featureX2featureY(featureX_oid, featureY_oid, association_type, attribute1, …, attributeN)*. Suitable indexes were created on all table attributes involved in SQL join or where clauses. For each of the three SQL strategies, we tested memory usage and running time performances (over 5 not subsequent runs, to avoid caching influence) in extracting a new association data table (*ResTab*) with an increasing number of attributes (Table A1). In doing so, we considered 3, 8 and 13 attributes, where 3 is the minimum number of attributes describing the features involved in a transitive relationship, and additional attributes are useful to better characterize the new association data identified. All tests were performed on a computer with Intel Core 2 Quad 2.66 GHz CPU, 12 GB RAM, 1 TB Sata II hard disk and Win 7 Ultimate

Edition (64 bit) operating system, giving a total Windows Experience Index of 5.9 (on a range from 1.0 to 7.9) [A2].

Benchmarking results

We tested and compared the three possible SQL strategies that can be used to perform the transitive relationship approach on large annotation data sets, as defined in the *Implementation of the transitive relationship approach* section above. Table A1 illustrates the obtained performance results of the three strategies in identifying missing associations and extracting an increasing number of attributes, which describe the characteristics of the features and their associations involved in the identified transitive relationships. Results indicate the strategy III as the one with always the best performance, regardless of the number of extracted attributes (average improvement in speed was of 59.31 % and 11.99 % with respect to the strategy I and II, respectively). Running times to extract an increasing number of attributes are fairly similar within each strategy, whereas the amount of required memory obviously grows (especially when attributes with textual descriptions are also taken, in addition to the initially extracted attributes with feature IDs). Yet, the time that would be required to subsequently extract these attributes, which contain information useful for the characterization and interpretation of the results, would be very high. Thus, the optimal choice resulted using the strategy III with the extraction of the full set of useful attributes. This choice optimizes the global performance, although with higher memory usage: SQL statements that extract fewer attributes are faster and require less memory, but later processing might be much slower. Thus, it is well suited to perform efficiently and effectively the transitive relationship approach on large annotation data sets stored in any relational database.

References

A1.Lu H, Mikkilineni KP, Richardson JP: **Design and evaluation of algorithms to compute the transitive closure of a database relation**. In: *Proc. Third IEEE Int. Conf. Data Eng.* Washington, DC, IEEE Computer Society 1987, 112-119.

A2.Microsoft Windows: **Windows Experience Index: Windows 7 features**. [Online]. 2014. Available from: http://windows.microsoft.com/en-US/windows7/products/features/windows-experience-index

Figures

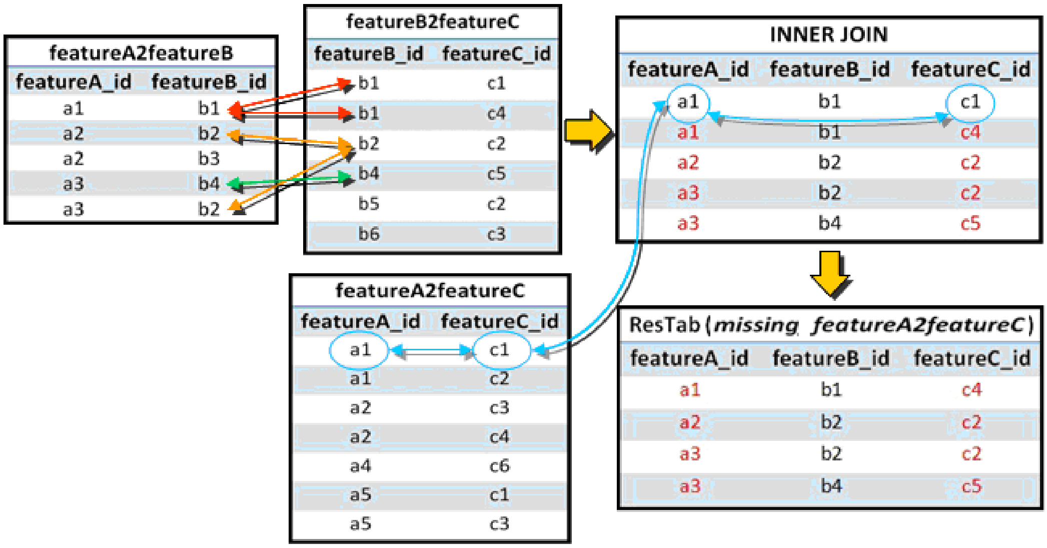

Figure A1: Discovery of missing associations by transitive relationship.

The new associations detected are in red, as it appears from comparison of the *INNER JOIN* table with the *featureA2featureC* table: the selected *a1* - *c1* ID pair is not identified since it is already present in the *featureA2featureC* table.

Tables

Table A1: Compared performance results of different SQL strategies for the transitive relationship approach

	**Strategy I**			**Strategy II**			**Strategy III**		
	**CREATE TABLE *ResTab* AS SELECT … FROM *featureA2featureB* AS FAB INNER JOIN *featureB2featureC* AS FBC USING (*featureB_oid*)** **WHERE (FAB.*featureA_oid*, FBC.*featureC_oid*) NOT IN (SELECT *featureA_oid*, *featureC_oid* FROM *featureA2featureC* AS FAC);**			**CREATE TABLE *ResTab* AS SELECT … FROM *featureA2featureB* AS FAB INNER JOIN *featureB2featureC* AS FBC USING (*featureB_oid*);** **DELETE FROM *ResTab* WHERE (*featureA_oid*, *featureC_oid*) IN (SELECT *featureA_oid*, *featureC_oid* FROM *featureA2featureC* AS FAC);**			**CREATE TABLE *ResTab* AS SELECT … FROM featureA2featureB AS FAB INNER JOIN *featureB2featureC* AS FBC USING (*featureB_oid*)** **LEFT JOIN *featureA2featureC* AS FAC USING (*featureA_oid*, *featureC_oid*) WHERE FAC.*featureA_oid* IS NULL;**		
**# of Attributes Extracted**	**3 Attributes**	**8 Attributes**	**13 Attributes**	**3 Attributes**	**8 Attributes**	**13 Attributes**	**3 Attributes**	**8 Attributes**	**13 Attributes**
**Average Time (s)**	11,175	11,998	12,099	5,077	5,421	5,830	4,594	4,822	4,932
**Standard Deviation Time (s)**	197.36	215.99	305.17	64.89	66.61	115.89	79.69	81.71	78.72
**Extracted Table Size (GB)**	2.31	2.85	3.68	2.31	2.85	3.68	2.31	2.85	3.68
**# of New Associations (Rows) Identified**	35,357,827								

Average and standard deviation times are over 5 not subsequent runs (to avoid caching influence)
