# Supplementary material for: Detection of gene annotations and protein-protein interaction associated disorders through transitive relationships between integrated annotations
Source: BMC Genomics. 2015 Jun 1;16(Suppl 6):S5. doi: 10.1186/1471-2164-16-S6-S5 (PMC4460591; doi:10.1186/1471-2164-16-S6-S5)
Supplement: Additional file 2 — Genetic disorders with potential associations with PPIs detected by transitive relationship. [file 1471-2164-16-S6-S5-S2.pdf]

Genetic disorders with potentential associations with PPIs detected by transitive relationship

Genetic Disorder	***PPI Associations***
ALZHEIMER DISEASE	72
BREAST CANCER	67
BARDET-BIEDL SYNDROME	48
HUNTINGTON DISEASE	28
DIABETES MELLITUS, NONINSULIN-DEPENDENT	27
COLORECTAL CANCER	23
MITOCHONDRIAL COMPLEX I DEFICIENCY	23
SYSTEMIC LUPUS ERYTHEMATOSUS	22
GLIOMA SUSCEPTIBILITY 1	18
INFLAMMATORY BOWEL DISEASE 1	18
LEUKEMIA, ACUTE MYELOID	18
PARKINSON DISEASE, LATE-ONSET	18
HEPATOCELLULAR CARCINOMA	17
FANCONI ANEMIA, COMPLEMENTATION GROUP D2	16
LUNG CANCER	13
RHEUMATOID ARTHRITIS	12
AMYOTROPHIC LATERAL SCLEROSIS 1	10
HERMANSKY-PUDLAK SYNDROME 1	9
LEIGH SYNDROME	9
PHOTOPAROXYSMAL RESPONSE	9
PROSTATE CANCER	9
BLOOM SYNDROME	8
ECTODERMAL DYSPLASIA, HYPOHIDROTIC, WITH IMMUNE DEFICIENCY	8
OVARIAN CANCER	8
CYSTIC FIBROSIS	7
SPINOCEREBELLAR ATAXIA 1	7
ALZHEIMER DISEASE 3	6
FANCONI ANEMIA, COMPLEMENTATION GROUP C	6
HEPATITIS C VIRUS, SUSCEPTIBILITY TO	6
LYNCH SYNDROME I	6
MULTIPLE SCLEROSIS, SUSCEPTIBILITY TO	6
MUSCULAR DYSTROPHY, DUCHENNE TYPE	6
PICK DISEASE OF BRAIN	6
SCHIZOPHRENIA	6
CORNELIA DE LANGE SYNDROME 1	5
DIABETES MELLITUS, INSULIN-DEPENDENT	5
ECTODERMAL DYSPLASIA 1, HYPOHIDROTIC, X-LINKED	5
HUMAN IMMUNODEFICIENCY VIRUS TYPE 1, SUSCEPTIBILITY TO	5
HYPERCHOLESTEROLEMIA, FAMILIAL	5
LI-FRAUMENI SYNDROME 1	5
SCLERODERMA, FAMILIAL PROGRESSIVE	5
ATAXIA-TELANGIECTASIA	4
ECTODERMAL DYSPLASIA, ANHIDROTIC, WITH IMMUNODEFICIENCY, OSTEOPETROSIS	4
EXUDATIVE VITREORETINOPATHY 1	4
FRONTOTEMPORAL DEMENTIA	4
INCONTINENTIA PIGMENTI	4
MARFAN SYNDROME	4
MYCOBACTERIUM TUBERCULOSIS, SUSCEPTIBILITY TO	4
NEUROFIBROMATOSIS, TYPE I	4
NIJMEGEN BREAKAGE SYNDROME	4

OSTEOGENIC SARCOMA	4
PARKINSON DISEASE 8, AUTOSOMAL DOMINANT	4
PSORIASIS SUSCEPTIBILITY 1	4
WERNER SYNDROME	4
ABDOMINAL OBESITY-METABOLIC SYNDROME	3
ACUTE PROMYELOCYTIC LEUKEMIA	3
ASTHMA, SUSCEPTIBILITY TO	3
ATYPICAL MYCOBACTERIOSIS, FAMILIAL	3
AUTOIMMUNE LYMPHOPROLIFERATIVE SYNDROME	3
BARE LYMPHOCYTE SYNDROME, TYPE I	3
BERNARD-SOULIER SYNDROME	3
BURKITT LYMPHOMA	3
CEREBRAL CAVERNOUS MALFORMATIONS	3
DENTATORUBRAL-PALLIDOLUYSIAN ATROPHY	3
DESMOID DISEASE, HEREDITARY	3
ERYTHROCYTOSIS, FAMILIAL, 2	3
EWING SARCOMA	3
FAMILIAL ADENOMATOUS POLYPOSIS 1	3
FANCONI ANEMIA, COMPLEMENTATION GROUP A	3
FIBRODYSPLASIA OSSIFICANS PROGRESSIVA	3
GASTROINTESTINAL STROMAL TUMOR	3
LEUKEMIA, ACUTE LYMPHOBLASTIC	3
MISMATCH REPAIR CANCER SYNDROME	3
MITOCHONDRIAL COMPLEX IV DEFICIENCY	3
NOONAN SYNDROME	3
PARKINSON DISEASE 2, AUTOSOMAL RECESSIVE JUVENILE	3
PEUTZ-JEGHERS SYNDROME	3
RENAL CELL CARCINOMA, NONPAPILLARY	3
RETINOBLASTOMA	3
THYROID CARCINOMA, PAPILLARY	3
VON HIPPEL-LINDAU SYNDROME	3
XERODERMA PIGMENTOSUM, COMPLEMENTATION GROUP F	3
AFIBRINOGENEMIA, CONGENITAL	2
ALAGILLE SYNDROME 1	2
ATAXIA, EARLY-ONSET, WITH OCULOMOTOR APRAXIA AND HYPOALBUMINEMIA	2
ATAXIA-TELANGIECTASIA-LIKE DISORDER	2
BRACHYDACTYLY, TYPE A2	2
C1Q DEFICIENCY	2
CELIAC DISEASE	2
DEMENTIA, LEWY BODY	2
DIGEORGE SYNDROME OR VELOCARDIOFACIAL SYNDROME	2
DOWN SYNDROME	2
EPIDERMOLYSIS BULLOSA JUNCTIONALIS WITH PYLORIC ATRESIA	2
FANCONI ANEMIA, COMPLEMENTATION GROUP E	2
GASTRIC CANCER	2
GASTRIC CANCER, HEREDITARY DIFFUSE	2
GRANULOMATOUS DISEASE, CHRONIC, AUTOSOMAL RECESSIVE, CYTOCHROME B-POSITIVE	2
HEMOCHROMATOSIS	2
HUTCHINSON-GILFORD PROGERIA SYNDROME	2
IGE RESPONSIVENESS, ATOPIC	2
JUVENILE POLYPOSIS SYNDROME	2

LEUKEMIA, CHRONIC LYMPHOCYTIC	2
LEUKEMIA, CHRONIC MYELOID	2
LONG QT SYNDROME 1	2
LYMPHOPROLIFERATIVE SYNDROME, X-LINKED, 1	2
MACHADO-JOSEPH DISEASE	2
MAJOR AFFECTIVE DISORDER 1	2
MEDULLOBLASTOMA	2
MENTAL RETARDATION, X-LINKED, SYNDROMIC 14	2
MICROVASCULAR COMPLICATIONS OF DIABETES, SUSCEPTIBILITY TO, 1	2
MICROVASCULAR COMPLICATIONS OF DIABETES, SUSCEPTIBILITY TO, 4	2
MOHR-TRANEBJAERG SYNDROME	2
MUSCULAR DYSTROPHY, CONGENITAL MEROSIN-DEFICIENT, 1A	2
MYOTONIC DYSTROPHY 1	2
NARCOLEPSY 1	2
NAXOS DISEASE	2
NERVE GROWTH FACTOR, ALPHA SUBUNIT	2
PFEIFFER SYNDROME	2
PHEOCHROMOCYTOMA	2
PITUITARY ADENOMA, ACTH-SECRETING	2
POLYCYSTIC KIDNEY DISEASE 1	2
PULMONARY HYPERTENSION, PRIMARY, 1	2
RENAL CELL CARCINOMA, XP11-ASSOCIATED	2
SPINAL AND BULBAR MUSCULAR ATROPHY, X-LINKED 1	2
SPINAL MUSCULAR ATROPHY, TYPE I	2
SPINOCEREBELLAR ATAXIA 7	2
TANGIER DISEASE	2
TESTICULAR GERM CELL TUMOR	2
WILLIAMS-BEUREN SYNDROME	2
XERODERMA PIGMENTOSUM, COMPLEMENTATION GROUP A	2
XERODERMA PIGMENTOSUM, COMPLEMENTATION GROUP E	2
ACROFACIAL DYSOSTOSIS 1, NAGER TYPE	1
ALOPECIA AREATA 1	1
ALPHA-THALASSEMIA	1
AMYLOIDOSIS, FAMILIAL VISCERAL	1
ARRHYTHMOGENIC RIGHT VENTRICULAR DYSPLASIA, FAMILIAL, 12	1
ARRHYTHMOGENIC RIGHT VENTRICULAR DYSPLASIA, FAMILIAL, 2	1
ARTHROGRYPOSIS, RENAL DYSFUNCTION, AND CHOLESTASIS 1	1
BASAL CELL NEVUS SYNDROME	1
BECKWITH-WIEDEMANN SYNDROME	1
BLADDER CANCER	1
BLOOD GROUP--KELL SYSTEM	1
BRACHYDACTYLY, TYPE C	1
BRANCHIOOTORENAL SYNDROME 1	1
CAMURATI-ENGELMANN DISEASE	1
CARDIOFACIOCUTANEOUS SYNDROME	1
CARDIOMYOPATHY, DILATED, 1GG	1
CEREBRAL AMYLOID ANGIOPATHY, APP-RELATED	1
CEREBRAL ARTERIOPATHY, AUTOSOMAL DOMINANT, WITH SUBCORTICAL INFARCTS	1
CEREBRAL CAVERNOUS MALFORMATIONS 2	1
CHARGE SYNDROME	1
CHONDROSARCOMA, EXTRASKELETAL MYXOID	1

CHROMOSOME 5Q DELETION SYNDROME	1
CLEIDOCRANIAL DYSPLASIA	1
COCKAYNE SYNDROME, TYPE A	1
COCKAYNE SYNDROME, TYPE B	1
COFFIN-SIRIS SYNDROME	1
COMPLEMENT COMPONENT C1R/C1S DEFICIENCY	1
CORNEAL DYSTROPHY, FUCHS ENDOTHELIAL, 1	1
CORNELIA DE LANGE SYNDROME 2	1
COWDEN SYNDROME 1	1
COWDEN SYNDROME 2	1
CREUTZFELDT-JAKOB DISEASE	1
CUTIS LAXA, AUTOSOMAL RECESSIVE, TYPE IA	1
DIAMOND-BLACKFAN ANEMIA	1
DIHYDROPYRIMIDINASE DEFICIENCY	1
DYSKERATOSIS CONGENITA, X-LINKED	1
DYSTONIA 1, TORSION, AUTOSOMAL DOMINANT	1
ECTODERMAL DYSPLASIA 10A, HYPOHIDROTIC/HAIR/NAIL TYPE, AUTOSOMAL DOMINANT	1
ECTODERMAL DYSPLASIA 10B, HYPOHIDROTIC/HAIR/TOOTH TYPE, AUTOSOMAL	1
EMERY-DREIFUSS MUSCULAR DYSTROPHY 1, X-LINKED	1
ENDOMETRIAL CANCER	1
EPIDERMOLYSIS BULLOSA SIMPLEX, AUTOSOMAL RECESSIVE	1
EPIDERMOLYSIS BULLOSA SIMPLEX, DOWLING-MEARA TYPE	1
EPIDERMOLYSIS BULLOSA SIMPLEX, GENERALIZED	1
EPIDERMOLYSIS BULLOSA SIMPLEX, LOCALIZED	1
ERYTHROCYTOSIS, FAMILIAL, 3	1
EXUDATIVE VITREORETINOPATHY 2, X-LINKED	1
FACIOSCAPULOHUMERAL MUSCULAR DYSTROPHY 1	1
FACTOR V AND FACTOR VIII, COMBINED DEFICIENCY OF, 1	1
FACTOR V AND FACTOR VIII, COMBINED DEFICIENCY OF, 2	1
FANCONI ANEMIA, COMPLEMENTATION GROUP F	1
FRIEDREICH ATAXIA 2	1
FRONTOTEMPORAL LOBAR DEGENERATION WITH TDP43 INCLUSIONS, GRN-RELATED	1
FUNDUS DYSTROPHY, PSEUDOINFLAMMATORY, OF SORSBY	1
GILLES DE LA TOURETTE SYNDROME	1
GLANZMANN THROMBASTHENIA	1
GLANZMANN THROMBASTHENIA, AUTOSOMAL DOMINANT	1
GLAUCOMA, PRIMARY OPEN ANGLE	1
GLOMERULOPATHY WITH FIBRONECTIN DEPOSITS 2	1
GLUCOCORTICOID DEFICIENCY 1	1
GM1-GANGLIOSIDOSIS, TYPE I	1
GRAFT-VERSUS-HOST DISEASE, SUSCEPTIBILITY TO	1
GRISCELLI SYNDROME, TYPE 1	1
HEINZ BODY ANEMIAS	1
HELICOBACTER PYLORI INFECTION, SUSCEPTIBILITY TO	1
HEMANGIOMA, CAPILLARY INFANTILE	1
HEMORRHAGE, INTRACEREBRAL, SUSCEPTIBILITY TO	1
HEREDITARY LEIOMYOMATOSIS AND RENAL CELL CANCER	1
HETEROTOPIA, PERIVENTRICULAR, X-LINKED DOMINANT	1
HISTIOCYTOMA, ANGIOMATOID FIBROUS	1
HYPERFERRITINEMIA WITH OR WITHOUT CATARACT	1
HYPER-IGE RECURRENT INFECTION SYNDROME, AUTOSOMAL DOMINANT	1

HYPERLIPIDEMIA, FAMILIAL COMBINED	1
HYPERLIPOPROTEINEMIA, TYPE I	1
HYPERTENSION, ESSENTIAL	1
HYPOALPHALIPOPROTEINEMIA, PRIMARY	1
HYPOGONADOTROPIC HYPOGONADISM 16 WITH OR WITHOUT ANOSMIA	1
HYPOGONADOTROPIC HYPOGONADISM 2 WITH OR WITHOUT ANOSMIA	1
HYPOMAGNESEMIA 3, RENAL	1
HYPOTRICHOSIS 1	1
ICHTHYOSIS, CONGENITAL, AUTOSOMAL RECESSIVE 2	1
ICHTHYOSIS, CONGENITAL, AUTOSOMAL RECESSIVE 3	1
IMMUNODEFICIENCY 11; IMD11	1
INSENSITIVITY TO PAIN, CONGENITAL, WITH ANHIDROSIS	1
INTRINSIC FACTOR DEFICIENCY	1
INVASIVE PNEUMOCOCCAL DISEASE, RECURRENT ISOLATED, 1	1
IRAK4 DEFICIENCY	1
JERVELL AND LANGE-NIELSEN SYNDROME 1	1
JUVENILE MYELOMONOCYTIC LEUKEMIA	1
KABUKI SYNDROME 1	1
KERATOSIS, SEBORRHEIC	1
KLEEFSTRA SYNDROME	1
KLEINE-LEVIN HIBERNATION SYNDROME	1
LACRIMOAURICULODENTODIGITAL SYNDROME	1
LEBER CONGENITAL AMAUROSIS 1	1
LEUKOENCEPHALOPATHY WITH VANISHING WHITE MATTER	1
LI-FRAUMENI SYNDROME 2	1
LOEYS-DIETZ SYNDROME, TYPE 1A	1
LOWE OCULOCEREBRORENAL SYNDROME	1
LYMPHANGIOLEIOMYOMATOSIS	1
LYMPHOMA, NON-HODGKIN, FAMILIAL	1
MACULAR DEGENERATION, AGE-RELATED, 1	1
MACULAR DEGENERATION, AGE-RELATED, 2	1
MAPLE SYRUP URINE DISEASE	1
MATURITY-ONSET DIABETES OF THE YOUNG, TYPE 2	1
MECKEL SYNDROME, TYPE 1	1
MEGALENCEPHALY-CAPILLARY MALFORMATION-POLYMICROGYRIA SYNDROME	1
MEGALENCEPHALY-POLYMICROGYRIA-POLYDACTYLY-HYDROCEPHALUS SYNDROME	1
MEGALOBLASTIC ANEMIA 1	1
MELANOMA, CUTANEOUS MALIGNANT, SUSCEPTIBILITY TO, 1	1
MELANOMA, CUTANEOUS MALIGNANT, SUSCEPTIBILITY TO, 3	1
MELANOMA, UVEAL	1
MEMBRANOUS NEPHROPATHY, SUSCEPTIBILITY TO	1
MITOCHONDRIAL COMPLEX II DEFICIENCY	1
MUIR-TORRE SYNDROME	1
MULTIPLE ACYL-COA DEHYDROGENASE DEFICIENCY	1
MULTIPLE ENDOCRINE NEOPLASIA, TYPE I	1
MULTIPLE ENDOCRINE NEOPLASIA, TYPE IIA	1
MULTIPLE ENDOCRINE NEOPLASIA, TYPE IIB	1
MUSCULAR DYSTROPHY, LIMB-GIRDLE, TYPE 2C	1
MUSCULAR DYSTROPHY-DYSTROGLYCANOPATHY (CONGENITAL WITH BRAIN AND EYE	1
MYELODYSPLASTIC SYNDROME	1
MYOCARDIAL INFARCTION, SUSCEPTIBILITY TO	1

MYOCLONIC EPILEPSY OF LAFORA	1
MYOPATHY, MYOFIBRILLAR 6, MFM6	1
MYOPATHY, TUBULAR AGGREGATE	1
MYOTONIC DYSTROPHY 2	1
NEMALINE MYOPATHY 2	1
NEPHRONOPHTHISIS 1	1
NEUTROPENIA, NONIMMUNE CHRONIC IDIOPATHIC, OF ADULTS	1
NORRIE DISEASE	1
OBESITY	1
OCULAR CICATRICIAL PEMPHIGOID	1
OPTIC ATROPHY 2	1
OPTIC ATROPHY 4	1
OROFACIAL CLEFT 1	1
OROFACIAL CLEFT 11	1
OSTEOARTHRITIS SUSCEPTIBILITY 1	1
OSTEOMYELITIS, STERILE MULTIFOCAL, WITH PERIOSTITIS AND PUSTULOSIS	1
PANCREATIC CANCER	1
PARAGANGLIOMA AND GASTRIC STROMAL SARCOMA	1
PARAGANGLIOMAS 4	1
PARAGANGLIOMAS 5	1
PARKINSON-DEMENTIA SYNDROME	1
PAROTID PROLINE-RICH SALIVARY PROTEIN PC	1
PEMPHIGUS VULGARIS, FAMILIAL	1
PODOCONIOSIS, SUSCEPTIBILITY TO	1
POLYCYSTIC LIVER DISEASE	1
POLYCYTHEMIA VERA	1
PORENCEPHALY 1	1
PREMATURE OVARIAN FAILURE 1	1
PROTEUS SYNDROME	1
PSORIASIS SUSCEPTIBILITY 4	1
PULMONARY DISEASE, CHRONIC OBSTRUCTIVE	1
PYOGENIC STERILE ARTHRITIS, PYODERMA GANGRENOSUM, AND ACNE	1
RETINITIS PIGMENTOSA 2	1
RHABDOID TUMOR PREDISPOSITION SYNDROME 1	1
ROBERTS SYNDROME	1
ROTHMUND-THOMSON SYNDROME	1
SARCOIDOSIS, SUSCEPTIBILITY TO, 1	1
SCHISTOSOMA MANSONI INFECTION, SUSCEPTIBILITY/RESISTANCE TO	1
SEVERE COMBINED IMMUNODEFICIENCY, AUTOSOMAL RECESSIVE, T CELL-NEGATIVE	1
SEVERE COMBINED IMMUNODEFICIENCY, X-LINKED	1
SEVERE CUTANEOUS ADVERSE REACTION, SUSCEPTIBILITY TO	1
SICKLE CELL ANEMIA	1
SILVER-RUSSELL SYNDROME	1
SITOSTEROLEMIA	1
SJOGREN SYNDROME	1
SPASTIC PARAPLEGIA 4, AUTOSOMAL DOMINANT	1
SPASTIC PARAPLEGIA 5A, AUTOSOMAL RECESSIVE	1
SPERMATOGENIC FAILURE, Y-LINKED, 2	1
SPINOCEREBELLAR ATAXIA 2	1
SPONDYLOCOSTAL DYSOSTOSIS 1, AUTOSOMAL RECESSIVE	1
STREPTOCOCCUS, GROUP A, SEVERITY OF INFECTION BY	1

SUPRANUCLEAR PALSY, PROGRESSIVE, 1	1
TELANGIECTASIA, HEREDITARY HEMORRHAGIC, OF RENDU, OSLER, AND WEBER	1
THYROID CARCINOMA, FOLLICULAR	1
TRANSFERRIN SERUM LEVEL QUANTITATIVE TRAIT LOCUS 2	1
TRICHOTHIODYSTROPHY, PHOTOSENSITIVE	1
USHER SYNDROME, TYPE IC	1
VENTRICULAR TACHYCARDIA, CATECHOLAMINERGIC POLYMORPHIC, 1, WITH OR	1
VITAMIN D-DEPENDENT RICKETS, TYPE 2A	1
VITREORETINOPATHY, NEOVASCULAR INFLAMMATORY	1
WEST NILE VIRUS, SUSCEPTIBILITY TO	1
WISKOTT-ALDRICH SYNDROME	1
ZINC, ELEVATED PLASMA	1
